# Age-Dependent Effects of Chronic Stress on Zebrafish Behavior and Regeneration

**DOI:** 10.3389/fphys.2022.856778

**Published:** 2022-04-29

**Authors:** Angie Henríquez Martínez, Laura C. Ávila, María A. Pulido, Yeferzon A. Ardila, Veronica Akle, Natasha I. Bloch

**Affiliations:** ^1^ Department of Biomedical Engineering, University of Los Andes, Bogotá, Colombia; ^2^ School of Medicine, University of Los Andes, Bogotá, Colombia

**Keywords:** tissue regeneration, aging, zebrafish, behavior, anxiety, chronic stress (or “stress”)

## Abstract

Stress can have a significant impact on many aspects of an organism’s physiology and behavior. However, the relationship between stress and regeneration, and how this relationship changes with age remains poorly understood. Here, we subjected young and old zebrafish to a chronic stress protocol and evaluated the impact of stress exposure on multiple measures of zebrafish behavior, specifically thigmotaxis (open field test) and scototaxis (light/dark preference test), and on regeneration ability after partial tail amputation. We found evidence that young and older adult fish are differentially impacted by stress. Only young fish showed a significant change in anxiety-like behaviors after being exposed to chronic stress, while their regeneration ability was not affected by the stress protocol. On the other hand, older fish regenerated their caudal fin significantly slower compared to young fish, but their behavior remained unaffected after being exposed to stress. We further investigated the expression of two candidate genes (*nlgn1* and *sam2*) expressed in the central nervous system, and known to be associated with stress and anxiety-like behavior. The expression of stress-related gene candidate *sam2* increased in the brain of older individuals exposed to stress. Our results suggest there is a close relationship between chronic stress, regeneration, and behavior in zebrafish (*Danio rerio*), and that the impact of stress is age-dependent.

## Introduction

Physiological balance can be temporarily disrupted by external conditions including stressors. Stress responses are a crucial adaptation for proper resource allocation toward cognitive and muscular performance. Depending on the duration of the exposure to stressful stimuli, stress can be considered acute or chronic. Despite the adaptive value of stress responses, the prolonged activation of the corresponding pathways caused by chronic stress can negatively impact different physiological processes such as digestion, growth, reproduction, immune response, wound healing, and tissue regeneration (Sallin and Jaźwińska, 2016).

These biological processes are not only influenced by the external environment, but also by intrinsic factors such as age. Aging is a complex phenomenon that affects multiple aspects of an organism’s fitness and condition, from the cellular to the organismal level (Kishi et al., 2009). Aging has been demonstrated to reduce regeneration ability and cause cellular damage, including damage to DNA and proteins, leading to cognitive decline, circadian dysregulation and sleep disruption, among other alterations (Yu et al., 2006). As a consequence of age-dependent macromolecular damage and physiological function decline, behavior changes can be found between age cohorts, showing a decrease in locomotor activity and a differential response to novel situations (Shoji et al., 2016). Despite the growing research on age-dependent processes, their complex relationship with external factors, such as stress, remains poorly understood.

The physiological response to stressors involves alterations at the molecular level that can impact regeneration. Stressors are external stimuli that translate into nervous system signals that trigger the hypothalamus-pituitary-interrenal axis (HPI, in zebrafish) among other pathways (Alsop and Vijayan, 2009; Blum and Begemann, 2012, 2015; Wehner et al., 2014). This axis is responsible for controlling the levels of cortisol in the fish’s body and activates nuclear transcription factors that regulate genes related to glucose metabolism, immune function, and behavior (Alsop and Vijayan, 2009; Piato et al., 2011). When the organism is exposed to glucocorticoids for prolonged periods of time, regeneration processes can become impaired. This phenomenon was demonstrated in mice, where exposure to glucocorticoids compromised bone regeneration (Hachemi et al., 2018). Similarly, in zebrafish this exposure provoked a significant decrease in cardiac regeneration ability, angiogenesis, and cell proliferation (Huang et al., 2013). Additionally, increasing glucocorticoid levels can impair blastema formation after caudal fin amputations (Mathew et al., 2007).

The physiological alterations caused by stress, particularly chronic stress, involve gene expression changes along the pathways linked to stress responses. This molecular response starts with the perception of stress and gene expression changes in the brain. In order to fully understand how stress can impact physiology and behavior, we need to identify the genes responsible for triggering the stress response following stress perception in the brain. Candidate genes, such as *neuroligin 1* (*nlgn1*) and *samdori 2* (*sam2*)*,* have been shown to mediate adaptive responses to stress, anxiety, neurodegeneration and aging in different species, including zebrafish (Kilaru et al., 2016; Geng and Peterson, 2019). SAM2 is a chemokine expressed exclusively in the central nervous system, secreted as a chemo-attractant to guide the migration of cells of the immune system, and it has been also associated with neuroinflammatory responses (Rosténe et al., 2007; Choi et al., 2018). Its connection with the HPA axis was shown through repression of the corticotropin releasing hormone gene (*crh*) and possible neuromodulatory functions (Choi et al., 2018). Neuroligin 1 (NLGN-1) is a synaptic cell-adhesion protein that modulates the glutaminergic synapse. NLGN-1 has been associated with post-traumatic stress disorder (PTSD) in humans (Kilaru et al., 2016). Moreover, its variation was related to a predisposition to higher levels of anxiety or fear in mice, and it is important in the associative memory of fear in adult rats (Kim et al., 2008; Bian et al., 2019). Studying expression changes in these gene candidates in response to stress can help us make hypotheses about the beginning stages of the underlying mechanisms linking stress response to physiological changes and regeneration.

Animal models have contributed to our understanding of the physiological response to stressors, regeneration mechanisms and the genetic underpinnings of senescence and lifespan. Zebrafish is an excellent model to study processes related to stress, anxiety, and other neuropathologies due to its well-defined behavioral phenotypes and the existing paradigms to study them in the laboratory (Egan et al., 2009; Kalueff et al., 2014; Choi et al., 2018). Moreover, zebrafish possess evolutionarily conserved pathways present in other vertebrates and its neuroendocrine system provides robust physiological responses to stress, making its study relevant to our general understanding of stress responses (Alsop and Vijayan, 2008). The axis of stress regulation is highly conserved across vertebrates, from the HPA axis in mammals to the hypothalamus-pituitary–interrenal (HPI) axis in fish (Steenbergen et al., 2011). As a teleost, zebrafish is a common model for the study of regeneration due to its ability to regenerate many tissues and organs including fins, heart, kidney, and muscles, among others (Nakatani et al., 2007; Gemberling et al., 2013). In addition, zebrafish show gradual senescence like humans (Kishi et al., 2009), making them an outstanding animal model to study aging.

Despite a growing body of literature on stress and aging, there’s limited knowledge on the age-dependent effects of stress on behavior and regeneration. Even if there is ample research on individual factors related to stress, integrative approaches to understand the consequences of stress at different levels and at different ages remain scarce. Here we studied the effects of chronic stress exposure on behavior and regeneration at different ages. We further examined the expression of two gene candidates, *sam2* and *nlgn1*. Our study contributes to the understanding of the impact that stress has on an organism and how it changes with age.

## Materials and Methods

### Animals and Housing Conditions

We conducted all experiments in zebrafish, *Danio rerio wild type* TAB strain. We used a total of 48 older adult zebrafish, (3 years old; mixed females and males), and 52 young adult fish (10 months old; mixed females and males). Zebrafish populations were housed at the Laboratory of Neuroscience and Circadian Rhythms at the University of Los Andes, Colombia, under optimal conditions for the species (Harper and Lawrence, 2011). Fish were housed in a multi-tank re-circulating water system (Aquaneering, San Diego, CA, United States), on a 14:10 light/dark cycle and a temperature of 27.5 ± 0.5°C with salinity and pH in the range of 600–700 μS/cm and 7.2–7.8, respectively. Fish were fed twice a day with rearing food Aquatox Fish Diet of Zeigler Bros, Inc., United States and enriched with live brine shrimp (*Artemia salina* *—*INVE Aquaculture Nutrition, United States).

### Experimental Design

**FIGURE 1:**
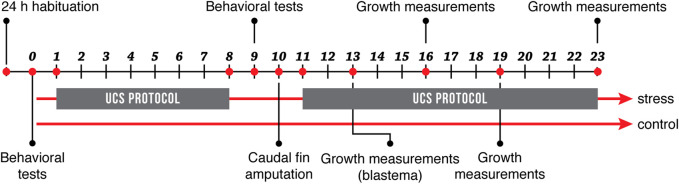
Experimental design. Fish in the stress group were exposed to an 8-day stress protocol (UCS), while fish in the control group remained under standard conditions. All fish were subjected to behavioral tests before and after the stress protocol. On day 10, caudal fins were amputated, and growth measurements were recorded on days 13, 16, 19, and 23.

Animals of both sexes within age groups were pooled and randomly assigned to two experimental groups: Control and Stress. At the beginning of the experiment, both experimental groups remained in separate tanks under standard conditions (as described above) during 24 h, for habituation. Fish in the stress group were exposed to an unpredictable chronic stress protocol (UCS) that lasted 8 days as described below (Piato et al., 2011; Manuel et al., 2014; Marcon et al., 2016, 2018; Rambo et al., 2017). From now on we will refer to this protocol as the stress protocol. Before beginning the stress protocol, we subjected all animals to open field test and a light/dark preference test to evaluate their baseline behavior. After the stress protocol, we repeated the same behavioral tests for all fish in the experiment (Figure 1). At the end of behavioral testing (day 10), we amputated the ventral distal part of the caudal fin in all animals to evaluate tissue regeneration, restarting the stress protocol for fish in the stress group until the end of the experiment. We registered the growth of the caudal fin by measuring tail length, on days 13, 16, 19, and 23 as described in Figure 1 and registering growth relative to the total caudal fin length of each fish. This experiment was done in 6 cohorts: three for old fish (n:20, n:20, n:12) and three for young fish (n:20, n:20, n:8).

### Unpredictable Chronic Stress Protocol

The stress protocol applied here is an adaptation of previous studies (Piato et al., 2011; Manuel et al., 2014; Marcon et al., 2016, 2018; Rambo et al., 2017). After habituation, fish in the stress group were exposed twice a day for 8 days (day 1 thru 8) to one of the following stressors: 1) heating tank water up to 33°C for 30 min; 2) cooling tank water up to 23°C for 30 min; 3) crowding of 10 animals for 40 min in a 250 ml beaker; 4) indirect exposure to a predator, *Archocentrus nigrofasciatus*, for 50 min avoiding direct contact; 5) low water level until animals’ dorsal body wall were exposed, for 2 min; 6) tank change, three consecutive times; 7) chasing animals for 8 min with a net. To maintain unpredictability, time and sequence of stressors’ presentation were randomized and changed daily (Supplementary Table S1). The different stressors were applied to all animals in the stress group at the same time of the day. The control group remained undisturbed throughout the entire duration of the experiment, unaffected by the procedures done to the stress group. As indicated in Figure 1, the stress protocol was initially performed in days 1 through 8 of the experiment for fish in the stress group and was then restarted and maintained for an additional 13 days after caudal fin amputation (day 11). Fish were under constant observation and no signs of severe suffering were detected. There was no mortality associated with the experiment. All fish had a normal appearance and locomotor activity in their home tanks. There were no unexpected events related to fish housing or feeding.

### Apparatus

The apparatus used in all behavioral tests was the same as the one described in (Maximino et al., 2010a). The tank (15 cm × 22.5 cm × 45 cm) was constructed with transparent acrylic and coated with black and white matte adhesive vinyl (to avoid shoaling tendencies due to following their own reflections), and a central sliding door to divide the tank in half. The water column was at 10 cm and the water was changed after each individual fish test. Behavioral tests were recorded using a webcam placed on top of the tank (Supplementary Figure S1). All the video files were analyzed with the video tracking software EthoVision XT 14 (Noldus Information Technology, Wageningen, Netherlands).

### Behavioral Tests

1) Open field test: for this test, we used only the white compartment of the tank (15 cm × 22.5 cm × 22.5 cm; Supplementary Figure S1). Each zebrafish was placed individually in the tank. Animals were recorded for 6 min to evaluate and analyze exploration and thigmotaxis (here understand as the fish’s tendency to stay close to the tank walls). The following variables were measured: total distance traveled (cm) and distance to center point of the arena (cm).
2) Light-dark preference test: each zebrafish was placed individually in the tank, with access to both compartments (black and white zones; Supplementary Figure S1). Animals were recorded for 10 min to evaluate scototaxis. The following variables were measured: total distance traveled (cm), time spent in the white zones (s) and total number of transitions between zones.

### Regeneration Ability

Once the stress protocol was completed, we photographed all fish from a side position and amputated the ventral part of the caudal fin of all animals using a Nikon AZ100 multizoom microscope, in both the stress and control groups as shown in (Supplementary Figure S1). To perform the amputation of the caudal fin, fish were anesthetized with 250 mg/L tricaine (MS-222) following standard protocols. When fish no longer had a motor response and the frequency of operculum movement has decreased, the caudal fin was cut using a scalpel as indicated in (Supplementary Figure S1). After amputation, fish were returned to their tank to recover from anesthesia. We recorded the growth of the caudal fin of each fish at 3, 6, 9, and 13 days post-amputation (dpa, on days 13, 16, 19, and 23 of the experiment). We carried out all measurements using features implemented in the microscope’s software (NIS-Elements). To normalize growth measurements, we calculated a regeneration index by dividing the length of the regenerated tissue by the initial length of the caudal fin of each fish.

### Gene Expression

Based on preliminary results of the regeneration process after exposure to stress, we evaluated the expression of *nlgn1* and *sam2* in older individuals, 12 days after amputation when the regeneration differences were the largest between treatments. Euthanasia was carried out by an overdose of tricaine. We collected heads in 2 ml of RNAlater and stored at −20°C until processing (control: *n* = 5; stress group: *n* = 4). The brains were dissected out under a stereoscope (Nikon SMZ745) and re-stored in RNAlater at −20°C. Total brain RNA was then extracted with Aurum ™ Total RNA Mini Kit (BIO-RAD, United States). The integrity, purity and concentration of RNA were confirmed by gel electrophoresis and NanoDrop measurements (ND-2000 NanoDrop spectrophotometer). cDNA synthesis was carried out with iScript Select cDNA Synthesis Kit (BIO-RAD, United States). We then used qRT-PCR to quantify the relative expression of *nlgn1* and *sam2,* together with housekeeping genes (*ef1a* and *rpl13a*) as endogenous controls. We ran focal genes and housekeeping genes in parallel for all samples, using iTaq ™ universal SYBR^®^ Green supermix kit (BIO-RAD, United States) in a Qiagen qRT-PCR ROTOR-GENE Q machine (Qiagen, United States).

We analyzed fluorescence data from qRT-PCR runs with DART-PCR (Peirson et al., 2003). This method has been proven to be efficient and reliable to estimate the efficiency of amplification for each pair of primers from its amplification profile, thus allowing to estimate the fluorescence of each reaction duly corrected for the efficiency (Peirson et al., 2003; Bloch, 2014). The relative expression $R_{0}$ was calculated as follows:

$$R_{0}=\;R_{Ct}\times {\left(1+E\right)}^{-Ct}$$

Where Ct is the threshold cycle, $R_{Ct}$ is the fluorescence in this cycle and E is the amplification efficiency (Peirson et al., 2003). We calculated the relative expression of each gene using the average of the relative expression $\;R_{0}$ of the housekeeping genes as:

$$Normalized\;R_{0\;}=\frac{Targetgene\;R_{0\;}}{\left(ef1aR_{0\;}+rpl13a\;R_{0\;}\;\right)/2}$$

The Normalized Ro was multiplied by 100 to express target gene expressions as a percentage of housekeeping genes expression. Details of primers design and sequences can be found in supplementary materials (Supplementary Table S2).

### Statistical Analysis

After verifying normality with Q-Q plot for each variable in R, results of the behavioral tests were analyzed using Welch’s t-test to compare stress and control groups of each age group. Results of regeneration test were analyzed using a linear mixed model (LMM), that incorporates repeated measures for each growth measurement. Here, we used growth of the amputated section as dependent variable, total tail length as a covariate (to normalize for overall tail size), the interaction between measurement record and treatment as fixed effect, and the cohort as a random factor. This model allowed us to evaluate whether regeneration changes between treatments at each measurement point. Gene expression results were analyzed using Welch’s t-test to compare stress and control groups. Differences were considered significant with a threshold of *p* < 0.05, except for specific exceptions specified along the text. All results are expressed as the mean ± standard error of mean (S.E.M). Statistical analyses were performed in R, using packages lme4, nlme, FSA, dplyr, rstatix, ggpubr, lmtest, and Matrix.

## Results

### Behavior: Chronic Stress Exposure Induced Greater Changes in the Behavior of Young Fish Than in Old Zebrafish

**FIGURE 2:**
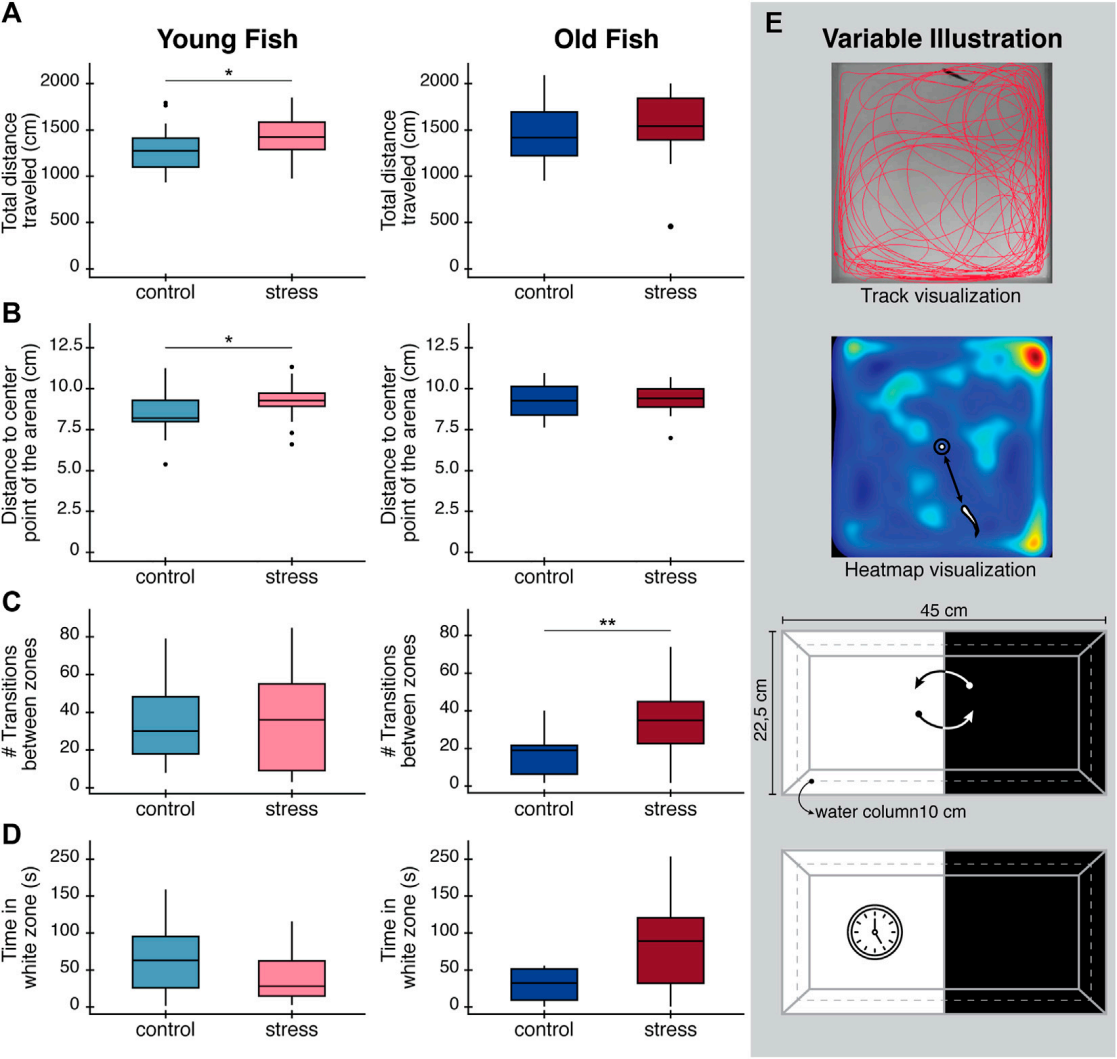
Effects in behavioral parameters (open field test and light/dark preference test) in zebrafish after exposure to a chronic stress protocol at day 9. **(A)** Total distance traveled in the open field test. **(B)** Distance to the center of the arena to evaluate thigmotaxis. **(C)** Total number of transitions between white and black zones during light/dark preference test. **(D)** Total time spent in the white zone of the tank during light/dark preference test as an exploratory behavior. Boxplots in panels **(A–D)**, are drawn such that the upper and lower whiskers limit interquartile range (Q1, Q3) and the line represents the median (Q2). **(E)** Diagrams illustrating variable being measured in each case. Top: track visualization used to obtain the total distance traveled of zebrafish. Middle: heatmap visualization and how distance to the center point of the arena was defined. Bottom: Illustration of the number of transitions between the white and the black zones, and time spend in the white zone only. All results are expressed as the mean ± standard error of mean (S.E.M). Statistical analysis corresponds to Welch´s t-test. *p < 0.05; **p < 0.01; ***p < 0.001.

In the open field test, young stressed fish traveled significantly longer distances (*p* = 0.04, Table 1; Figure 2A) compared to young unstressed controls. Young fish exposed to the stress protocol also showed a clear preference for the periphery of the tank, indicated by the greater distance between the animals and the center of the arena (*p* = 0.04, Table 1; Figure 2B), compared to control fish of the same age. In contrast, we found no significant differences between older zebrafish exposed to the stress protocol and controls for any of these variables (Table 1; Figure 2). However, it is worth noting that there was a baseline difference between young and older zebrafish, with control older animals swimming longer distances than control young animals, albeit with borderline statistical significance (*p* = 0.06, Supplementary Table S3).

**TABLE 1:** Results of statistical analyses between experimental groups (control, stress) before and after the stress protocol.

	T-test control vs. stress								
		Before stress protocol				After stress protocol			
		Control mean ± SE	Stress mean ± SE	t (df)	*p*-value	Control mean ± SE	Stress mean ± SE	t (df)	*p*-value
Open field	**Distance to the center point of the arena**								
	Young	8.56 ± 0.27	8.79 ± 0.20	0.68 (42.47)	0.50	8.51 ± 0.25	9.22 ± 0.23	2.09 (45.36)	**0.042**
	Old	8.59 ± 0.40	8.22 ± 0.32	−0.71 (27.23)	0.49	9.23 ± 0.27	9.32 ± 0.23	0.25 (27.93)	0.80
	**Total distance moved**								
	Young	1,300.23 ± 58.29	1,353.23 ± 56.89	0.65 (45.97)	0.52	1,255.84 ± 69.63	1,427.76 ± 42.30	2.11 (37.94)	**0.042**
	Old	1,532.02 ± 101.60	1,557.66 ± 93.77	0.18 (28.62)	0.85	1,454.02 ± 87.40	1,519.52 ± 95.11	0.51 (28.93)	0.62
Light/dark preference	**Time in white zone**								
	Young	83.79 ± 11.84	52.19 ± 10.01	−2.04 (44.76)	**0.048**	89.42 ± 18.68	55.09 ± 11.31	−1.57 (37.87)	0.12
	Old	148.90 ± 61.15	95.0 ± 24.73	−0.82 (18.49)	0.42	98.07 ± 39.78	72.91 ± 13.60	−0.60 (17.24)	0.56
	**Total number of transitions between zones**								
	Young	35.00 ± 4.16	26.25 ± 3.89	−1.53 (45.79)	0.13	35.62 ± 4.17	38.33 ± 3.90	0.36 (41.74)	0.72
	Old	9.60 ± 3.38	34.31 ± 5.48	3.84 (24.73)	**<** **0.001**	18.87 ± 3.47	37.25 ± 4.85	3.08 (26.78)	**0.005**

All *p*-values correspond to statistical tests evaluating the significance of differences between experimental treatments, thus control versus stress groups. Bold indicate *p*-values < 0.05.

In the light/dark preference test, we found no statistical difference in the time spent in the white zone between control and stressed fish of either age group after performing the stress protocol (Table 1; Figure 2D)*.* Interestingly, at baseline (on day 0) we found statistically significant differences in the time spent in white zone for young fish (*p* = 0.048, Table 1) and statistically significant differences in the number of transitions between white/dark zones in the old fish group (*p* < 0.001, Table 1)*.* These differences in the baseline behavior of both age groups, before administering the stress protocol, make the results of these tests difficult to interpret.

### Regeneration: The Ability and Rate of Regeneration Differ Between Young and Old Zebrafish After Exposure to Chronic Stress

**FIGURE 3:**
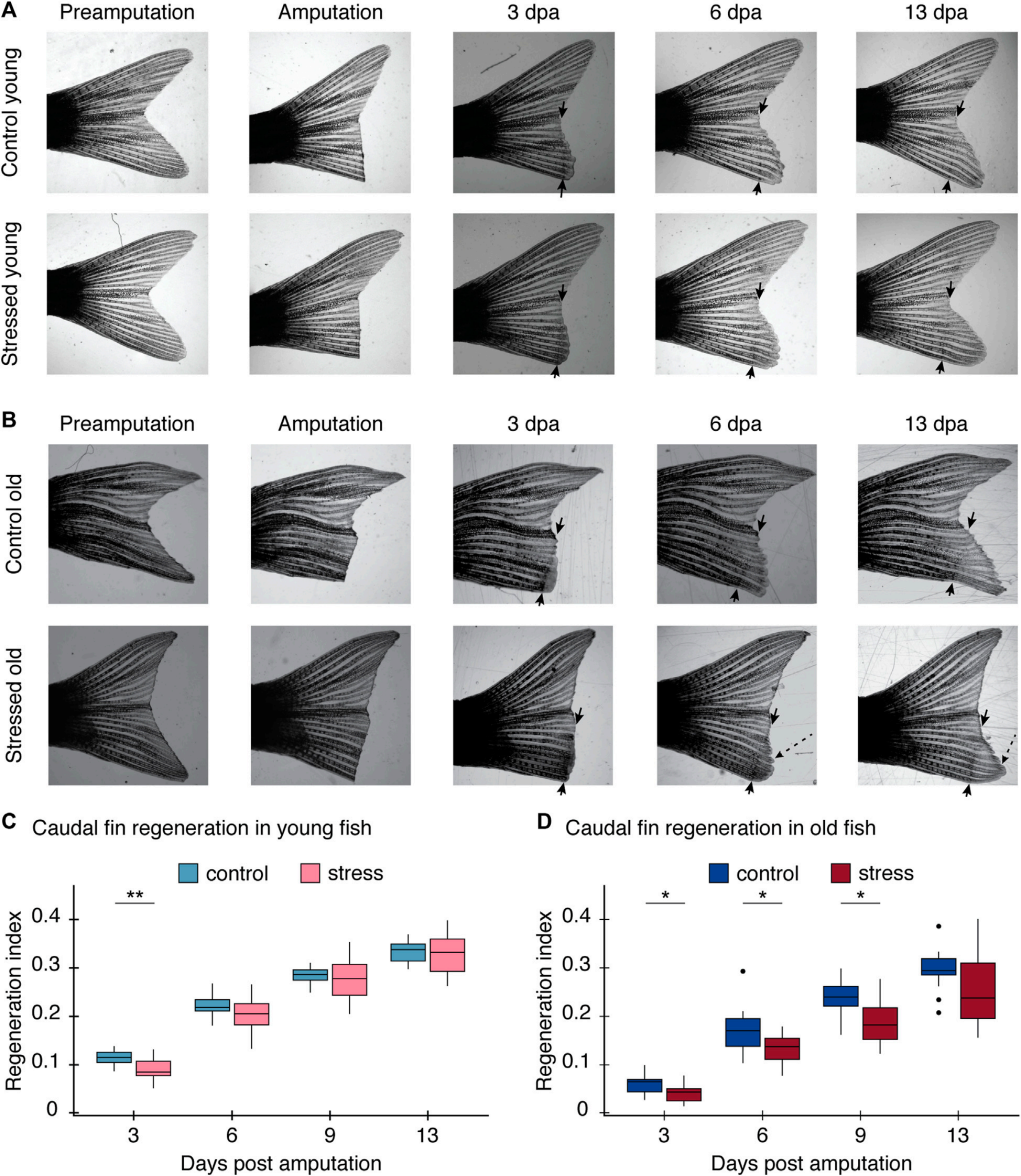
Regeneration process after the stress protocol. The regeneration index is calculated as the length of the regenerated tissue/initial length of the caudal fin of each fish measured from the most proximal part of the lepidotrichia. **(A)** Images of caudal fin growth measurements after amputation for young control fish (top row) and young stressed fish (bottom row). **(B)** Images of caudal fin growth measurements after amputation for old control fish (top row) and old stressed fish (bottom row). Black arrows indicate the amputation site. An additional measurement was done at 9 dpa but the image was excluded for clarity; complete regeneration process shown in Supplementary Figure S2 for young and Supplementary Figure S3 for old fish. Note heterogeneous growth in old fish (dashed line arrow). Further results on these unusual growth pattern can be seen in Supplementary Figure S4. **(C)** Graph comparing the regeneration index of the control and stress young caudal fin at 3, 6, 9, and 13 dpa; two cohorts included in this experiment. **(D)** Graph comparing the regeneration index of the control and stress old caudal fin at 3, 6, 9, and 13 dpa; two cohorts included in this experiment. All results are expressed as the mean ± standard error of mean (S.E.M). *p < 0.05; **p < 0.01; ***p < 0.001.

We found zebrafish regeneration is indeed disrupted by the exposure to chronic stress conditions (Figure 3). The caudal fin regenerated to full size in all fish, control and stressed in both age groups. However, the regeneration rate and pattern differed between experimental conditions. In young zebrafish, the effect of the stress protocol was only significant for the first measurement, at 3 days post amputation (dpa) (*p* = 0.003; Table 2; Figure 3B). All measurements thereafter of tail growth for young fish showed no significant effect of the stress protocol (6 dpa: *p* = 0.30, 9 dpa: *p* = 0.90, 13 dpa: *p* = 0.87, Table 2; Figure 3B), indicating that after 3 dpa, regeneration occurred in a normal way in young fish exposed to the stress protocol (Supplementary Figure S2). On the other hand, in old zebrafish we did observe a significant effect of the stress protocol on regeneration. The length of the regenerated tissue in the stressed group was significantly shorter than in the control group at 3, 6, and 9 dpa in old fish (3 dpa: *p* = 0.02*,* 6 dpa: *p* = 0.04, 9 dpa: *p* = 0.012, Table 2; Figure 3C). Even if the rate of regeneration was slower in stressed old fish, by the end of our experiment, at 13 dpa, they had fully regenerated their tails (Supplementary Figure S3) and we no longer found differences between control and stressed fish (13 dpa: *p* = 0.10, Table 2; Figure 3C)*.* Hence, with the regeneration test we identified a stronger impact of chronic stress in the early stages of the regeneration process in both age groups.

**TABLE 2:** Caudal fin growth measurements after amputation for young and old zebrafish.

	3 dpa		6 dpa		9 dpa		13 dpa	
	Mean ± SE	*p*-Value	Mean ± SE	*p*-Value	Mean ± SE	*p*-Value	Mean ± SE	*p*-Value
Young								
Control	0.60 ± 0.02	**0.003**	1.16 ± 0.04	0.30	1.51 ± 0.05	0.90	1.79 ± 0.06	0.87
Stress	0.27 ± 0.03		1.10 ± 0.04		1.50 ± 0.06		1.77 ± 0.05	
Old								
Control	0.40 ± 0,04	**0.02**	1.09 ± 0.09	**0.04**	1.54 ± 0.09	**0.012**	1.91 ± 0.13	0.10
Stress	0.27 ± 0.03		0.86 ± 0.05		1.22 ± 0.07		1.62 ± 0.12	

Bold indicate *p*-values < 0.05

In addition to the differences we found in the regeneration rate in both groups, we found differences in the patterns in which the regenerative process occurred after stress exposure, particularly in the older age groups. At the beginning of the regeneration process at 3 dpa, we observed a delay in blastema formation in stressed old fish (Figure 3A; Table 2). Then, after 13 dpa, the caudal fin of old zebrafish had grown back shorter and often with an irregular shape (Supplementary Figure S3, Supplementary Figure S4). In contrast, in the same time span, the caudal fins of young fish had regained their original shape and size (Figure 3, Supplementary Figure S2)*.* These results suggest a relationship between the age of the zebrafish and the ability to regenerate tissue after the exposure to chronic stress conditions.

After amputation in young fish, we observed three animals (3/10) with wounds in the growing caudal fin after the stress protocol, a potential sign of aggression and further impact of stress on young fish behavior (Supplementary Figure S5). Nevertheless, these young individuals were able to regrow the wounded tissue and completed the regeneration process, ending with functional caudal fins with similar shape and size to the original tails in the period of the experiments.

### Gene Expression: The Expression of *sam2* and *nlgn1* Increased in Old Zebrafish Exposed to the Stress Protocol

**FIGURE 4:**
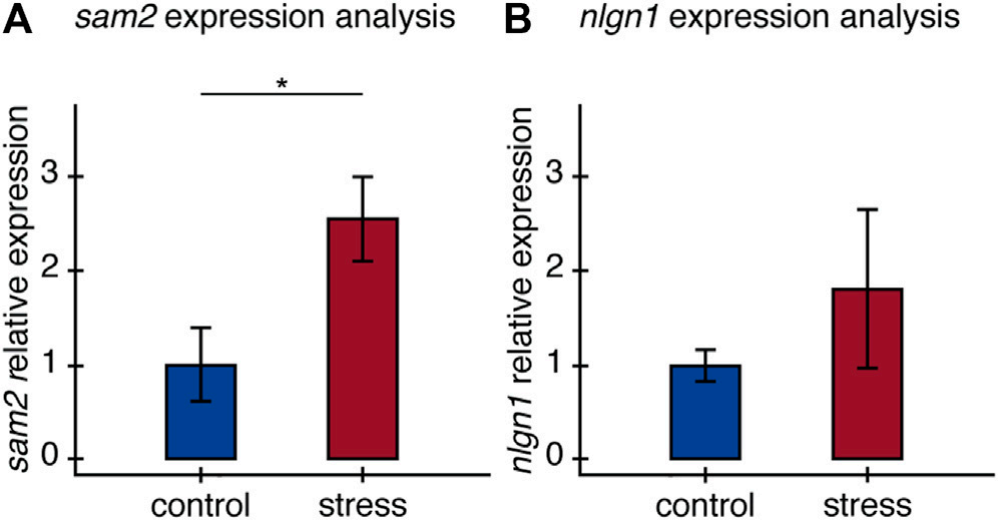
Relative expression of **(A)** *sam2* and **(B)** *nlgn1* in the brain of old fish. Relative expression of *sam2* and *nlgn1* between control and stress groups, calculated as fold-change differences in expression to the average expression of two housekeeping genes All results are expressed as the mean ± standard error: *p < 0.05.

Relative expression of *sam2* and *nlgn1* was estimated by normalizing to two housekeeping genes. We found older fish exposed to stress had significantly higher *sam2* expression than fish in the control group. Relative expression of *sam2* increased by 2.55 ± 0.45-fold in individuals exposed to the stress protocol (*t* = −2.42, *p* = 0.045) (Figure 4A). For *nlgn1* on the other hand, we did not observe significant differences in relative expression between experimental groups (*t* = −1.22, *p* = 0.26), despite a clear trend for stressed fish to express *nlgn1* at higher levels (Figure 4B). The lack of statistical significance here can be explained by greater variance in the stressed group for the relative expression of this gene.

## Discussion

The response to stress can be an adaptative strategy essential for survival, competition, and general fitness. However, chronic activation of the physiological processes triggered by continuous chronic exposure to stress can be harmful. Several studies have evaluated the relationship between stress and regeneration (Sallin and Jaźwińska, 2016), regeneration and aging (Anchelin et al., 2011), stress and behavior (Evans, 2001; Guski, 2001; Schulte, 2013; Van der Kooij et al., 2014), and even behavioral stress responses and aging (Haigis and Yankner., 2010). However, to our knowledge, this is the first study investigating how chronic stress can differentially impact regeneration and behavior in fish of different ages. Using an integrative approach in zebrafish, we found that chronic stress affects tissue regeneration and behavior differently in young and older fish. Exposure to chronic stress had a stronger impact on young fish’s behavior, while we have no evidence that older fish behavior is altered by exposure to stress. We observed the opposite effect for regeneration, which was significantly slower in old fish exposed to stress, but was not affected in young fish. We also identified changes in the relative expression of *sam2* in the brain of older fish. Our results suggest that this gene can be considered a potential candidate for further study on the mechanisms behind the impact of stress on physiological processes.

Stress and anxiety-like responses depend on both extrinsic and intrinsic factors. Extrinsic factors like weather or predation risk, and intrinsic factors such as the age, may affect the baseline behavior of an individual (Schulte, 2013; Derenne and Baron, 2002; Shoji et al., 2016). Our results provide further evidence for the effect age can have on behavior as we find behavioral differences between young and old zebrafish. At baseline, even before exposure to stress, open field test results revealed younger fish traveled shorter distances (and at slower velocity) compared to older fish. This variable reflects higher exploratory behavior in older zebrafish (Supplementary Table S3). Our results are consistent with previous reports of behavioral and cognitive differences that arise with age in zebrafish (Yu et al., 2006) and mice (Shoji et al., 2016). Together with these results, our findings indicate that age indeed affects the behavior of the animal, and it is an important feature to consider when studying behavior.

In this study, we found that exposure to chronic stress triggered changes in the behavior of zebrafish. These changes were age-dependent and altered zebrafish’s locomotor activity (Table 1)*.* As seen in track visualization and heatmaps (Supplementary Figure S6), stressed fish have an increased preference for corners and walls, probably looking for protection and shelter (Champagne et al., 2010). All these behavioral changes that arise after exposure to the stress protocol are consistent with anxiety-like behavioral patterns (Bailey and Crawley, 2009; Cachat et al., 2010). Interestingly, the effects of stress on behavior were different between age groups. In young zebrafish, we observed remarkable thigmotaxis after stress exposure, along with an increase in the total distance traveled and distance to the center point of the arena in the open field test. Zebrafish will typically spend time exploring the arena when exposed to a novel environment (Champagne et al., 2010). However, when anxious, fish will break this pattern by staying closer to the walls (thigmotaxis), similar to the behavior of humans exposed to anxiety and fear situations (Kallai et al., 2007). Despite not being statistically significant, young fish also seem to spend less time in the white area of the tank after being exposed to stress, further corroborating a tendency toward anxiogenic behaviors after being stressed in zebrafish (Maximino et al., 2010b) and rodents (Roth and Katz, 1979; Katz et al., 1981). In contrast, we found no evidence of anxiogenic behavioral markers in older fish, which continue to behave in a similar way to control fish after being exposed to the stress protocol. This differential impact of stress on behavior could be associated with structural and physiological changes in the brain that can reduce the autonomic responses and limit anxiety with aging as was demonstrated in humans and zebrafish (Flint et al., 2002; Yu et al., 2006).

The differential response to stress we observe between age groups can be explained by changes that occur in information processing as well as cognitive processes with aging. Changes in coping strategies and perception thresholds with age (Urry and Gross, 2010; Ebner and Fischer, 2014) can impact how stimuli are integrated (Shoji et al., 2016) and the effects of stressors in the organism (Sapolsky et al., 1986; Novais et al., 2017; Zapater-Fajarí et al., 2021). Moreover, differences in the behavioral response to stress between age groups can be attributed to the older individuals' experience (Scott et al., 2013; Epel and Lithgow, 2014; Andrews et al., 2017). Experience can cause differentiated neurogenesis, specifically in the dentate gyrus in the hypothalamus, producing stress-adapted neurons that mediate the response to subsequent stressors. Experience can thus influence stimuli interpretation and behavioral outcomes resulting in behavioral resilience (Dranovsky and Leonardo, 2012; De Miguel et al., 2018). The age-dependent effect of stress on behavior we observe is also consistent with age differences in emotional regulation capacities reported in mice and humans (Urry and Gross, 2010; Ebner and Fischer, 2014; Shoji and Miyakawa, 2019). These studies showed older individuals have decreased fluctuations in behavioral outputs after stress or exposure to a new environment, compared to younger ones, which is consistent with our findings.

In addition to the effects on behavior, we saw that chronic stress exposure impairs zebrafish regeneration. Stressed zebrafish showed lower rate of tissue regeneration than control zebrafish (Figure 3). Early in the wound healing process, fish living in standard conditions (i.e., control animals) showed faster blastema growth the fish exposed to the stress protocol in both age groups. The blastema is a proliferative tissue with undifferentiated cells necessary for regeneration and axonal outgrowth (Akimenko et al., 2003; Wills et al., 2008; Sehring et al., 2016, Akle et al., 2012). Our results indicate stressed fish had completely regrown amputated fin tissues by 13 dpa, at which point the differences between stressed and control fish were no longer evident (Figure 3; Supplementary Figure S2, Supplementary Figure S3). This suggests a stronger effect of chronic stress on the early formation of regenerative tissue, the blastema. Our results are consistent with previous studies in which the effects of stress on zebrafish heart regeneration were evident in the initial stages of regeneration and then growth became progressively normal (Sallin and Jaźwińska, 2016).

Wound epidermis and blastema formation are mediated by many signaling pathways. We propose that exposure to the stress protocol may alter signaling processes and the initiation of regeneration by increasing glucocorticoid levels. Retinoic acid (RA) and insulin-like growth factor (IGF) signaling are two of the most important pathways in the initial part of the regenerative process, while fibroblast growth factors (Fgf) signaling is determinant during the regenerative outgrowth stage (Chablais and Jaźwińska, 2010; Blum and Begemann 2012; Wehner et al., 2014; Blum and Begemann, 2015; Lee et al., 2005; Sehring et al., 2016). We know that stress, which involves glucocorticoids release, causes variation in IGF-1 secretion (Hachemi et al., 2018), more specifically by downregulating the expression of *igf1*, relating to the neuroendocrine stress response and factors linked to growth in fish (Nakano et al., 2013). These effects have been previously demonstrated in zebrafish showing exposure to glucocorticoids provoked a significant decrease in cardiac regeneration, angiogenesis and cell proliferation (Huang et al., 2013), and impaired blastema formation (Mathew et al., 2007). We suggest that chronic stress conditions cause an unbalance in regeneration signaling delaying the rapid proliferation of cells and the formation of the blastema. Further experiments could address the effect of these signaling molecules in different aged animals during regeneration.

Once more, we found age-dependent effects in the impact of chronic stress on regeneration. Older fish showed a significant reduction in the capacity to grow back the amputated tissue, reflected on the rate at which they regenerated their fins after stress exposure. On the contrary, younger zebrafish under chronic stress conditions completely regrew the amputated tissue at the same rate as control fish (Figure 3). Our results are consistent with previous research in which Anchelin et al. (2011) found that 12 days after amputating part of the caudal fin, old zebrafish grew the tissue back at a rate 50% lower than younger zebrafish. In addition to the rate of regeneration, the morphology of regenerated tissue was also affected by stress in older fish. Even though the caudal fin grew back to its original size in older zebrafish at the end of our experiment, some subjects had a very irregular tail shape (16 out of 24 fish in the stress condition; Supplementary Figure S4). This irregular growth could reflect an unbalance of the mechanisms responsible for coordinating the regeneration of caudal fin bones, since each bone ray of the caudal fin can regenerate independently (Pfefferli and Jaźwińska, 2015). Added to these studies, our findings confirm the role age plays as a determinant factor in the response to stress.

Telomere length and telomerase activity could be at the basis of this age-dependent effect of stress on regeneration. Telomere length and telomerase activity have been shown to decrease with age (Anchelin et al., 2011) and can have a profound impact on regeneration (Carneiro et al., 2016; Opresko and Shay, 2017). In zebrafish, telomere length increases during development until 18 months and starts decreasing drastically as fish become older at 24 months old (Anchelin et al., 2011). Based on this, we could argue that telomere length and development were therefore drastically different between our experimental groups, which were 10 and 36 months old, explaining the observed differences in regeneration ability.

Dissecting the molecular mechanisms linking stress responses in the brain to an organism’s behavior and physiology is key to understanding the stress response. We examined the expression of two candidate genes known to be associated with stress and expressed in the central nervous system, *sam2* and *nlgn1* (Kim et al., 2008; Choi et al., 2018). Examining the expression of these candidates’ genes can provide further evidence of their association with stress responses. Very little is known about the function of *sam2* in vertebrates, though its expression has been previously associated with the stress response axis, and the display of anxiety-like behavior in mice and zebrafish (Choi et al., 2018). Choi and colleagues observed increased anxiety-related responses in zebrafish and mouse *sam2* knockout models and found *sam2* repressed *Corticotropin-releasing hormone* (*crh*) expression and thus the HPA axis. These findings lead us to predict *sam2* expression would increase in fish after stress exposure as a modulator of *crh* expression. Our results are consistent with this prediction, as *sam2* expression was significantly higher in older fish exposed to chronic stress. In the future, it would be interesting to expand this experiment to examine the expression of the candidate genes at different ages.

The association we found between *sam2* expression levels and stress exposure suggest this gene may be part of the stress response in the brain, justifying further work to better understand its role, mechanisms of action and link to physiological processes such as regeneration. Previous work, although limited can help us formulate hypotheses on the potential role of *sam2* mediating stress responses. The link between *sam2* and the HPA axis revealed by Choi et al. (2018) allow us to speculate about the mechanisms that could link *sam2* to physiological processes. Mainly, based on their results the authors suggest that *sam2* could regulate CRH neuron activity in the PVN and is thus responsible for triggering gene expression pathways associated with the stress response (Aguilera and Liu, 2012; Choi et al., 2018). They further show SAM2 is involved in tonic GABAergic suppression of the CRH pathway. In addition to Choi et al. findings, elevated activity of glucocorticoid signaling was shown to cause a pro-inflammatory adult response impacting immunoregulation and regenerative capacity (Hartig et al., 2016). The downstream effects of CRH activation and the corresponding glucocorticoid release could ultimately link *sam2* expression to regeneration (Hartig et al., 2016). Taking into account the impact of stress on regeneration, the link between *sam2* and the suppression of the CRH pathway (Aguilera and Liu, 2012; Choi et al., 2018), and the effects of glucocorticoid signaling on immunoregulation and regenerative capacity (Hartig et al., 2016), we believe studying the link between *sam2* expression and regeneration is an interesting avenue for future research.

Neuroligin-1 (*nlgn1*) on the other hand, is a synaptic cell-adhesion molecule, involved in the formation of CNS synapses related, among other functions, to the perception of environmental stimuli (Kim et al., 2008; Xu et al., 2009; Kilaru et al., 2016). In general terms, *nlgn1* has been linked with the response to stress and stress related disorders. Its variation has been associated with a predisposition to higher levels of anxiety or fear in mice, and it is important in the associative memory of fear in adult rats (Kim et al., 2008; Bian et al., 2019). While this gene has not been directly connected to regeneration or its related pathways, we hypothesized its expression would also increase in stressed fish, making it a valuable candidate gene. The relationship between *nlgn1* expression and stress, however, is not clear in the literature, as other studies have also reported no change in *nlgn1* expression after stress exposure (Van der Kooij et al., 2014). Despite the tendency we observe for increased *nlgn1* expression in stressed individuals, our results are not statistically significant and difficult to interpret in the framework of previous evidence. Thus, we cannot determine whether the change in *nlgn1* expression in our experiment is indeed related to stress exposure or if it's involved in mediating the stress response.

Overall, we found stress impacts zebrafish behavior and regeneration, and it does so in an age-dependent manner. While stress mainly affects young fish behavior, it impacts fish regeneration ability. Moreover, we suggest a role of *sam2* mediating stress perception in the brain and hypothesize this gene could trigger activity in the stress response pathways that may ultimately impact physiological processes in older fish. Our findings highlight the importance of understanding the relationship between environmental stimuli and physiological responses, and how they can change at different life stages. By investigating the effects of stress on regeneration, our research provides insights for further research on the design of medical treatments that consider the impact of stress on the recovery processes.

## Data Availability

The original contributions presented in the study are included in the article/Supplementary Material, further inquiries can be directed to the corresponding author.

## Supplementary Material

Supplementary Figure S1 Behavioral testing apparatus and caudal fin amputation. **(A)** Diagram of the tank used in the behavioral tests and the disposition of the camera for test recordings. **(B)** Graphic representation of the amputation performed on every zebrafish in the ventral part of the caudal fin.

Supplementary Figure S2 Regeneration processes after exposure to the stress protocol in young zebrafish. **(A)** Time-lapse images of the regeneration processes after the amputation of the lower part of the caudal fin in control (top rows) and stressed (bottom rows) young fish. Red arrows indicate the amputation site. **(B)** Comparison between the initial shape of the caudal fin (uncut) and the appearance of the same fin after 13 dpa in a control zebrafish and **(C)** in a stressed individual.

Supplementary Figure S3 Regeneration processes after exposure to the stress protocol in old zebrafish. **(A)** Time-lapse images of the regeneration processes after the amputation of the lower part of the caudal fin in a control (top row) and stressed (bottom row) old fish. Red arrows indicate the amputation site. **(B)** Comparison between the initial shape of the caudal fin (uncut) and the appearance of the same fin after 13 dpa in a control zebrafish and **(C)** in a stressed individual.

Supplementary Figure S4 Irregular tissue growing in stressed old fish. Caudal fins from a stressed old fish that show bites and signals of aggression after the UCS protocol and before amputation.

Supplementary Figure S5 Aggression signals after exposure to the stress protocol in young stressed fish. **(A–C)** Caudal fins from three different young stressed fish that show bites and signals of aggression after the UCS protocol and before amputation. We also show the complete regeneration of the caudal fins, both in the wounds and in the amputation.

Supplementary Figure S6 Visualization of the zebrafish behavior during Open Field test. Track visualization and heatmap visualization of the swimming patterns of three representative fish from each experimental group (control, stress) in **(A)** young and **(B)** old zebrafish. Rows are the same representative fish during the same recording.

Supplementary Table S1 Procedure of the unpredictable chronic stress (UCS) protocol in zebrafish.

Supplementary Table S2 Primer design. Target-specific primers for *sam2*, *elf-1*, and *rpl13a* were designed using primer blast and primer quest tools in conjunction with OligoAnalyzer program for the evaluation of the characteristics if each primer. Primers for *nlgn-1* were taken from Krall et al. (2019). These primers pairs amplify short fragments between 70–200 bp, in an exon-exon boundary to avoid amplification of possible contamination with genomic DNA and with Tm close to 60°C, considering a difference in Tm between the pair of primers 2°C.

Supplementary Table S3 Results of statistical analyses between age groups (young, old) before and after the stress protocol.

## References

[B1] Aguilera G. Liu Y. (2012). The Molecular Physiology of CRH Neurons. Front. Neuroendocrinology 33 (1), 67–84. 10.1016/j.yfrne.2011.08.002 PMC4341841 21871477

[B2] Akimenko M.-A. Marí-Beffa M. Becerra J. Géraudie J. (2003). Old Questions, New Tools, and Some Answers to the Mystery of Fin Regeneration. Dev. Dyn. 226 (2), 190–201. 10.1002/dvdy.10248 12557198

[B3] Akle V. Guelin E. Yu L. Brassard-Giordano H. Slack B. E. Zhdanova I. V. (2012). F-spondin/spon1b Expression Patterns in Developing and Adult Zebrafish. PLoS One 7 (6), e37593. 10.1371/journal.pone.0037593 22768035 PMC3387172

[B4] Alsop D. Vijayan M. M. (2008). Development of the Corticosteroid Stress Axis and Receptor Expression in Zebrafish. Am. J. Physiology-Regulatory, Integr. Comp. Physiol. 294 (3), R711–R719. 10.1152/ajpregu.00671.2007 18077507

[B5] Alsop D. Vijayan M. (2009). The Zebrafish Stress Axis: Molecular Fallout from the Teleost-specific Genome Duplication Event. Gen. Comp. Endocrinol. 161 (1), 62–66. 10.1016/j.ygcen.2008.09.011 18930731

[B6] Anchelin M. Murcia L. Alcaraz-Pérez F. García-Navarro E. M. Cayuela M. L. (2011). Behaviour of Telomere and Telomerase during Aging and Regeneration in Zebrafish. PLoS One 6 (2), e16955. 10.1371/journal.pone.0016955 21347393 PMC3036734

[B7] Andrews C. Nettle D. Larriva M. Gillespie R. Reichert S. Brilot B. O. (2017). A Marker of Biological Age Explains Individual Variation in the Strength of the Adult Stress Response. R. Soc. Open Sci. 4 (9), 171208. 10.1098/rsos.171208 28989794 PMC5627134

[B8] Bailey K. R. Crawley J. N. (2009). “Anxiety-Related Behaviors in Mice,” in Methods of Behavior Analysis in Neuroscience. 2nd ed. (Boca Raton, FL: CRC Press/Taylor & Francis), 77–95. PMID: 21204329. 21204329

[B9] Bian Y. Yang L. Zhao M. Li Z. Xu Y. Zhou G. (2019). Identification of Key Genes and Pathways in post-traumatic Stress Disorder Using Microarray Analysis. Front. Psychol. 10. 10.3389/fpsyg.2019.00302 PMC6403462 30873067

[B10] Bloch N. I. (2015). Evolution of Opsin Expression in Birds Driven by Sexual Selection and Habitat. Proc. R. Soc. B. 282, 20142321. 10.1098/rspb.2014.2321 PMC4262183 25429020

[B11] Blum N. Begemann G. (2012). Retinoic Acid Signaling Controls the Formation, Proliferation and Survival of the Blastema during Adult Zebrafish Fin Regeneration. Development 139 (1), 107–116. 10.1242/dev.065391 22096078

[B12] Blum N. Begemann G. (2015). Retinoic Acid Signaling Spatially Restricts Osteoblasts and Controls Ray-Interray Organization during Zebrafish Fin Regeneration. Development 142 (17), 2888–2893. 10.1242/dev.120212 26253402

[B13] Cachat J. Stewart A. Grossman L. Gaikwad S. Kadri F. Chung K. M. (2010). Measuring Behavioral and Endocrine Responses to novelty Stress in Adult Zebrafish. Nat. Protoc. 5 (11), 1786–1799. 10.1038/nprot.2010.140 21030954

[B14] Carneiro M. C. de Castro I. P. Ferreira M. G. (2016). Telomeres in Aging and Disease: Lessons from Zebrafish. Dis. Model. Mech. 9 (7), 737–748. 10.1242/dmm.025130 27482813 PMC4958310

[B15] Chablais F. Jaźwińska A. (2010). IGF Signaling between Blastema and Wound Epidermis Is Required for Fin Regeneration. Development 137 (6), 871–879. 10.1242/dev.043885 20179093

[B16] Champagne D. L. Hoefnagels C. C. M. de Kloet R. E. Richardson M. K. (2010). Translating Rodent Behavioral Repertoire to Zebrafish (Danio Rerio): Relevance for Stress Research. Behav. Brain Res. 214 (2), 332–342. 10.1016/j.bbr.2010.06.001 20540966

[B17] Choi J.-H. Jeong Y.-M. Kim S. Lee B. Ariyasiri K. Kim H.-T. (2018). Targeted Knockout of a Chemokine-like Gene Increases Anxiety and Fear Responses. Proc. Natl. Acad. Sci. U.S.A. 115, 5. 10.1073/pnas.1707663115 PMC5798319 29339520

[B18] De Miguel Z. Haditsch U. Palmer T. D. Azpiroz A. Sapolsky R. M. (2018). Adult-generated Neurons Born during Chronic Social Stress Are Uniquely Adapted to Respond to Subsequent Chronic Social Stress. Mol. Psychiatry 24, 1178–1188. 10.1038/s41380-017-0013-1 29311652

[B19] Derenne A. Baron A. (2002). Behavior Analysis and the Study of Human Aging. Behav. Analyst 25 (2), 151–160. 10.1007/bf03392054 PMC2731606 22478383

[B20] Dranovsky A. Leonardo E. D. (2012). Is There a Role for Young Hippocampal Neurons in Adaptation to Stress? Behav. Brain Res. 227 (2), 371–375. 10.1016/j.bbr.2011.05.007 21621559 PMC3529657

[B21] Ebner N. C. Fischer H. k. (2014). Emotion and Aging: Evidence from Brain and Behavior. Front. Psychol. 5, 996. 10.3389/fpsyg.2014.00996 25250002 PMC4158975

[B22] Egan R. J. Bergner C. L. Hart P. C. Cachat J. M. Canavello P. R. Elegante M. F. (2009). Understanding Behavioral and Physiological Phenotypes of Stress and Anxiety in Zebrafish. Behav. Brain Res. 205 (1), 38–44. 10.1016/j.bbr.2009.06.022 19540270 PMC2922906

[B23] Epel E. S. Lithgow G. J. (2014). Stress Biology and Aging Mechanisms: Toward Understanding the Deep Connection between Adaptation to Stress and Longevity. Journals Gerontol. Ser. A: Biol. Sci. Med. Sci. 69 (Suppl. 1), S10–S16. 10.1093/gerona/glu055 PMC4022128 24833580

[B24] Evans G. W. (2001). Crowding and Other Environmental Stressors. International Encyclopedia of the Social & Behavioral Sciences, 3018–3022. 10.1016/b0-08-043076-7/01806-4

[B25] Flint A. Bradwejn J. Vaccarino F. Gutkowska J. Palmour R. Koszycki D. (2002). Aging and Panicogenic Response to Cholecystokinin Tetrapeptide: an Examination of the Cholecystokinin System. Neuropsychopharmacology 27 (4), 663–671. 10.1016/S0893-133X(02)00330-5 12377403

[B26] Gemberling M. Bailey T. J. Hyde D. R. Poss K. D. (2013). The Zebrafish as a Model for Complex Tissue Regeneration. Trends Genet. 29 (11), 611–620. 10.1016/j.tig.2013.07.003 23927865 PMC3812420

[B27] Geng Y. Peterson R. T. (2019). The Zebrafish Subcortical Social Brain as a Model for Studying Social Behavior Disorders. Dis. Model. Mech. 12 (8), dmm039446. 10.1242/dmm.039446 31413047 PMC6737945

[B28] Guski R. (2001). Environmental Stress and Health. International Encyclopedia of the Social & Behavioral Sciences, 4667–4671. 10.1016/b0-08-043076-7/03832-8

[B29] Hachemi Y. Rapp A. E. Picke A.-K. Weidinger G. Ignatius A. Tuckermann J. (2018). Molecular Mechanisms of Glucocorticoids on Skeleton and Bone Regeneration after Fracture. J. Mol. Endocrinol. 61 (1), R75–R90. 10.1530/JME-18-0024 29588427 PMC5976078

[B30] Haigis M. C. Yankner B. A. (2010). The Aging Stress Response. Mol. Cell 40 (2), 333–344. 10.1016/j.molcel.2010.10.002 20965426 PMC2987618

[B31] Harper C. Lawrence C. (2011). The Laboratory Zebrafish. 1st ed. Florida, US: CRC Press.

[B32] Hartig E. I. Zhu S. King B. L. Coffman J. A. (20162016). Cortisol-treated Zebrafish Embryos Develop into Pro-inflammatory Adults with Aberrant Immune Gene Regulation. Biol. Open 5 (8), 1134–1141. 10.1242/bio.020065 PMC5004618 27444789

[B33] Huang W.-C. Yang C.-C. Chen I.-H. Liu Y.-M. L. Chang S.-J. Chuang Y.-J. (2013). Treatment of Glucocorticoids Inhibited Early Immune Responses and Impaired Cardiac Repair in Adult Zebrafish. PLoS ONE 8 (6), e66613. 10.1371/journal.pone.0066613 23805247 PMC3689762

[B34] Kallai J. Makany T. Csatho A. Karadi K. Horvath D. Kovacs-Labadi. B. (2007). Cognitive and Affective Aspects of Thigmotaxis Strategy in Humans. Behav. Neurosci. 121 (1), 21–30. 10.1037/0735-7044.121.1.21 17324048

[B35] Kalueff A. V. Stewart A. M. Gerlai R. (2014). Zebrafish as an Emerging Model for Studying Complex Brain Disorders. Trends Pharmacol. Sci. 35 (2), 63–75. 10.1016/j.tips.2013.12.002 24412421 PMC3913794

[B36] Katz R. J. Roth K. A. Carroll B. J. (1981). Acute and Chronic Stress Effects on Open Field Activity in the Rat: Implications for a Model of Depression. Neurosci. Biobehavioral Rev. 5 (2), 247–251. 10.1016/0149-7634(81)90005-1 7196554

[B37] Kilaru V. Iyer S. V. Almli L. M. Stevens J. S. Lori A. Jovanovic T. (2016). Genome-wide Gene-Based Analysis Suggests an Association between Neuroligin 1 (NLGN1) and post-traumatic Stress Disorder. Transl. Psychiatry 6, e820. 10.1038/tp.2016.69 27219346 PMC5070067

[B38] Kim J. Jung S.-Y. Lee Y. K. Park S. Choi J.-S. Lee C. J. (2008). Neuroligin-1 Is Required for normal Expression of LTP and Associative Fear Memory in the Amygdala of Adult Animals. Proc. Natl. Acad. Sci. U.S.A. 105 (26), 9087–9092. 10.1073/pnas.0803448105 18579781 PMC2449369

[B39] Kishi S. Slack B. E. Uchiyama J. Zhdanova I. V. (2009). Zebrafish as a Genetic Model in Biological and Behavioral Gerontology: Where Development Meets Aging in Vertebrates &ndash; A Mini-Review. Gerontology 55 (4), 430–441. 10.1159/000228892 19654474 PMC2820570

[B40] Lee Y. Grill S. Sanchez A. Murphy-Ryan M. Poss K. D. (2005). Fgf Signaling Instructs Position-dependent Growth Rate during Zebrafish Fin Regeneration. Development 132 (23), 5173–5183. 10.1242/dev.02101 16251209

[B41] Manuel R. Gorissen M. Zethof J. Ebbesson L. O. E. van de vis H. Flik G. (2014). Unpredictable Chronic Stress Decreases Inhibitory Avoidance Learning in Tuebingen Long-Fin Zebrafish (*Danio rerio* Hamilton): Stronger Effects in the Resting Phase Than in the Active Phase. J. Exp. Biol. 217, 3919–3928. 10.1242/jeb.109736 25267842

[B42] Marcon M. Herrmann A. P. Mocelin R. Rambo C. L. Koakoski G. Abreu M. S. (2016). Prevention of Unpredictable Chronic Stress-Related Phenomena in Zebrafish Exposed to Bromazepam, Fluoxetine and Nortriptyline. Psychopharmacology 233, 3815–3824. 10.1007/s00213-016-4408-5 27562666

[B43] Marcon M. Mocelin R. Benvenutti R. Costa T. Herrmann A. P. De Oliveira D. L. (2018). Environmental Enrichment Modulates the Response to Chronic Stress in Zebrafish. J. Exp. Biol. 221, 4. 10.1242/jeb.176735 29361609

[B44] Mathew L. K. Sengupta S. Kawakami A. Andreasen E. A. Löhr C. V. Loynes C. A. (2007). Unraveling Tissue Regeneration Pathways Using Chemical Genetics. J. Biol. Chem. 282 (48), 35202–35210. 10.1074/jbc.M706640200 17848559

[B45] Maximino C. de Brito T. M. Colmanetti R. Pontes A. A. A. de Castro H. M. de Lacerda R. I. T. (2010). Parametric Analyses of Anxiety in Zebrafish Scototaxis. Behav. Brain Res. 210 (1), 1–7. 10.1016/j.bbr.2010.01.031 20117146

[B46] Maximino C. Marques de Brito T. Dias C. A. G. d. M. Gouveia A. Morato S. (2010). Scototaxis as Anxiety-like Behavior in Fish. Nat. Protoc. 5, 209–216. 10.1038/nprot.2009.225 20134420

[B47] Nakano T. Afonso L. O. B. Beckman B. R. Iwama G. K. Devlin R. H. (2013). Acute Physiological Stress Down-Regulates mRNA Expressions of Growth-Related Genes in Coho Salmon. PLoS ONE 8, e71421. 10.1371/journal.pone.0071421 23990952 PMC3747168

[B48] Nakatani Y. Kawakami A. Kudo A. (2007). Cellular and Molecular Processes of Regeneration, with Special Emphasis on Fish Fins. Dev. Growth Differ. 49 (2), 145–154. 10.1111/j.1440-169X.2007.00917.x 17335435

[B49] Novais A. Monteiro S. Roque S. Correia-Neves M. Sousa N. (2017). How Age, Sex and Genotype Shape the Stress Response. Neurobiol. Stress 6, 44–56. 10.1016/j.ynstr.2016.11.004 28229108 PMC5314441

[B50] Opresko P. L. Shay J. W. (2017). Telomere-associated Aging Disorders. Ageing Res. Rev. 33, 52–66. 10.1016/j.arr.2016.05.009 27215853 PMC9926533

[B51] Peirson S. N. Butler J. N. Foster R. G. (2003). Experimental Validation of Novel and Conventional Approaches to Quantitative Real-Time PCR Data Analysis. Nucleic Acids Res. 31 (14), 73e–73. 10.1093/nar/gng073 PMC167648 12853650

[B52] Pfefferli C. Jaźwińska A. (2015). The Art of Fin Regeneration in Zebrafish. Regeneration 2 (2), 72–83. 10.1002/reg2.33 27499869 PMC4895310

[B53] Piato Â. L. Capiotti K. M. Tamborski A. R. Oses J. P. Barcellos L. J. G. Bogo M. R. (2011). Unpredictable Chronic Stress Model in Zebrafish (*Danio rerio*): Behavioral and Physiological Responses. Prog. Neuro-Psychopharmacology Biol. Psychiatry 35, 561–567. 10.1016/j.pnpbp.2010.12.018 21187119

[B54] Rambo C. L. Mocelin R. Marcon M. Villanova D. Koakoski G. de Abreu M. S. (2017). Gender Differences in Aggression and Cortisol Levels in Zebrafish Subjected to Unpredictable Chronic Stress. Physiol. Behav. 171, 50–54. 10.1016/j.physbeh.2016.12.032 28039073

[B55] Rostène W. Kitabgi P. Parsadaniantz S. M. (2007). Chemokines: a New Class of Neuromodulator? Nat. Rev. Neurosci. 8, 895–903. 10.1038/nrn2255 17948033

[B56] Roth K. A. Katz R. J. (1979). Stress, Behavioral Arousal, and Open Field Activity-A Reexamination of Emotionality in the Rat. Neurosci. Biobehavioral Rev. 3 (4), 247–263. 10.1016/0149-7634(79)90012-5 542239

[B57] Sallin P. Jaźwińska A. (2016). Acute Stress Is Detrimental to Heart Regeneration in Zebrafish. Open Biol. 6 (3), 160012. 10.1098/rsob.160012 27030176 PMC4821245

[B58] Sapolsky R. M. Krey L. C. McEwen B. S. (1986). The Neuroendocrinology of Stress and Aging: The Glucocorticoid Cascade Hypothesis*. Endocr. Rev. 7 (3), 284–301. 10.1210/edrv-7-3-284 3527687

[B59] Schulte P. M. (2014). What Is Environmental Stress? Insights from Fish Living in a Variable Environment. J. Exp. Biol. 217 (1), 23–34. 10.1242/jeb.089722 24353201

[B60] Scott S. B. Sliwinski M. J. Blanchard-Fields F. (2013). Age Differences in Emotional Responses to Daily Stress: The Role of Timing, Severity, and Global Perceived Stress. Psychol. Aging 28 (4), 1076–1087. 10.1037/a0034000 24364410 PMC3874135

[B61] Sehring I. M. Jahn C. Weidinger G. (2016). Zebrafish Fin and Heart: What's Special about Regeneration? Curr. Opin. Genet. Development 40, 48–56. 10.1016/j.gde.2016.05.011 27351724

[B62] Shoji H. Miyakawa T. (2019). Age‐related Behavioral Changes from Young to Old Age in Male Mice of a C57 BL/6J Strain Maintained under a Genetic Stability Program. Neuropsychopharmacol. Rep. 39, 100–118. 10.1002/npr2.12052 30816023 PMC7292274

[B63] Shoji H. Takao K. Hattori S. Miyakawa T. (2016). Age-related Changes in Behavior in C57BL/6J Mice from Young Adulthood to Middle Age. Mol. Brain 9, 11. 10.1186/s13041-016-0191-9 26822304 PMC4730600

[B64] Steenbergen P. J. Richardson M. K. Champagne D. L. (2011). The Use of the Zebrafish Model in Stress Research. Prog. Neuro-Psychopharmacology Biol. Psychiatry 35 (6), 1432–1451. 10.1016/j.pnpbp.2010.10.010 20971150

[B65] Urry H. L. Gross J. J. (2010). Emotion Regulation in Older Age. Curr. Dir. Psychol. Sci. 19, 352–357. 10.1177/0963721410388395

[B66] van der Kooij M. A. Fantin M. Kraev I. Korshunova I. Grosse J. Zanoletti O. (2014). Impaired Hippocampal Neuroligin-2 Function by Chronic Stress or Synthetic Peptide Treatment Is Linked to Social Deficits and Increased Aggression. Neuropsychopharmacol 39 (5), 1148–1158. 10.1038/npp.2013.315 PMC3957108 24213355

[B67] Wehner D. Cizelsky W. Vasudevaro M. D. Özhan G. Haase C. Kagermeier-Schenk B. (2014). Wnt/β-Catenin Signaling Defines Organizing Centers that Orchestrate Growth and Differentiation of the Regenerating Zebrafish Caudal Fin. Cell Rep. 6 (3), 467–481. 10.1016/j.celrep.2013.12.036 24485658

[B68] Wills A. A. Kidd A. R. Lepilina A. Poss K. D. (2008). Fgfs Control Homeostatic Regeneration in Adult Zebrafish Fins. Development 135 (1), 3063–3070. 10.1242/dev.024588 18701543 PMC2748931

[B69] Xu J. Xiao N. Xia J. (2009). Thrombospondin 1 Accelerates Synaptogenesis in Hippocampal Neurons through Neuroligin 1. Nat. Neurosci. 13 (1), 22–24. 10.1038/nn.2459 19915562

[B70] Yu L. Tucci V. Kishi S. Zhdanova I. V. (2006). Cognitive Aging in Zebrafish. PLoS ONE 1 (1), e14. 10.1371/journal.pone.0000014 17183640 PMC1762370

[B71] Zapater-Fajarí M. Crespo-Sanmiguel I. Pulopulos M. M. Hidalgo V. Salvador A. (2021). Resilience and Psychobiological Response to Stress in Older People: The Mediating Role of Coping Strategies. Front. Aging Neurosci. 13, 67. 10.3389/fnagi.2021.632141 PMC7937969 33692681

