# Supplementary material for: Age-Dependent Effects of Chronic Stress on Zebrafish Behavior and Regeneration
Source: Front Physiol. 2022 Apr 29;13:856778. doi: 10.3389/fphys.2022.856778 (PMC9106366; doi:10.3389/fphys.2022.856778)
Supplement: Supplementary file 2 [file Table1.DOCX]

***Supplementary Material***

**Age-dependent effects of chronic stress on zebrafish behavior and regeneration**

Angie Henríquez^1,2^, Laura C. Ávila^1,2^, María A. Pulido^1^, Yeferzon A. Ardila^1^, Veronica Akle^1^, Natasha I. Bloch^2*^

^1^ School of Medicine,  University of Los Andes, Bogotá, 111711, Colombia

^2^Department of Biomedical Engineering,  University of Los Andes, Bogotá, 111711, Colombia

*** Correspondence:** 
**Natasha Bloch** 
[**n.blochm@uniandes.edu.co**](mailto:email@uni.edu)

**This PDF file includes:**

1. Supplementary Figures

- Figures S1 to S6

1. Supplementary Tables

- Tables S1 to S3

1. Supplementary References
2. ***Supplementary Figures***

**Figure S1. Behavioral testing apparatus and caudal fin amputation.** a) Diagram of the tank used in the behavioral tests and the disposition of the camera for test recordings. b) Graphic representation of the amputation performed on every zebrafish in the ventral part of the caudal fin.


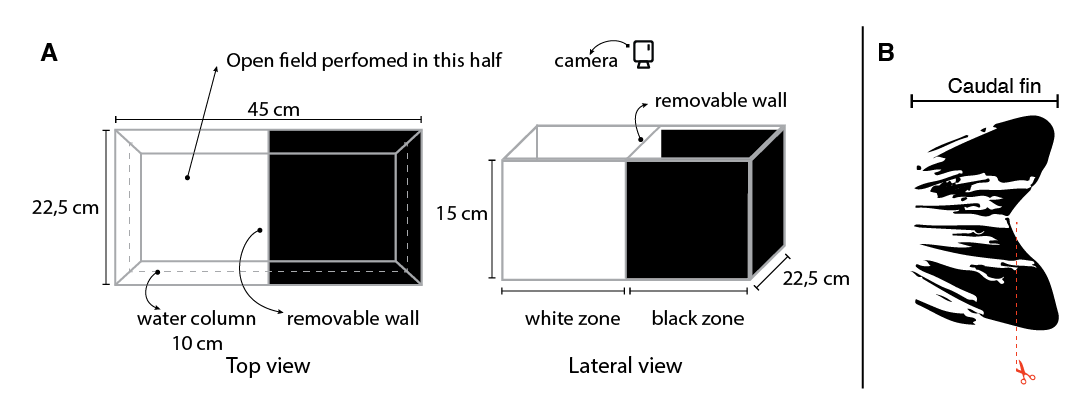


**Figure S2. Regeneration processes after exposure to the stress protocol in young zebrafish.** A) Time-lapse images of the regeneration processes after the amputation of the lower part of the caudal fin in control (top rows) and stressed (bottom rows) young fish. Red arrows indicate the amputation site. B) Comparison between the initial shape of the caudal fin (uncut) and the appearance of the same fin after 13 dpa in a control zebrafish and C) in a stressed individual.


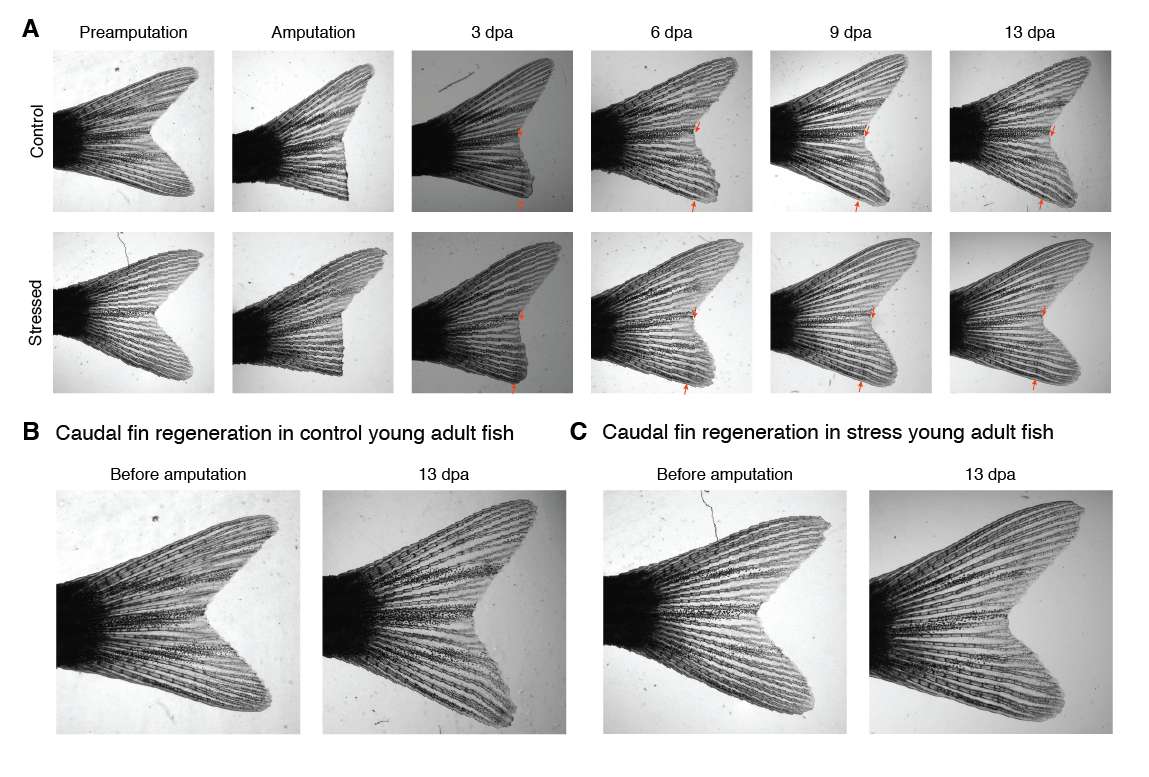


**Figure S3. Regeneration processes after exposure to the stress protocol in old zebrafish.** A) Time-lapse images of the regeneration processes after the amputation of the lower part of the caudal fin in a control (top row) and stressed (bottom row) old fish. Red arrows indicate the amputation site. B) Comparison between the initial shape of the caudal fin (uncut) and the appearance of the same fin after 13 dpa in a control zebrafish and C) in a stressed individual.


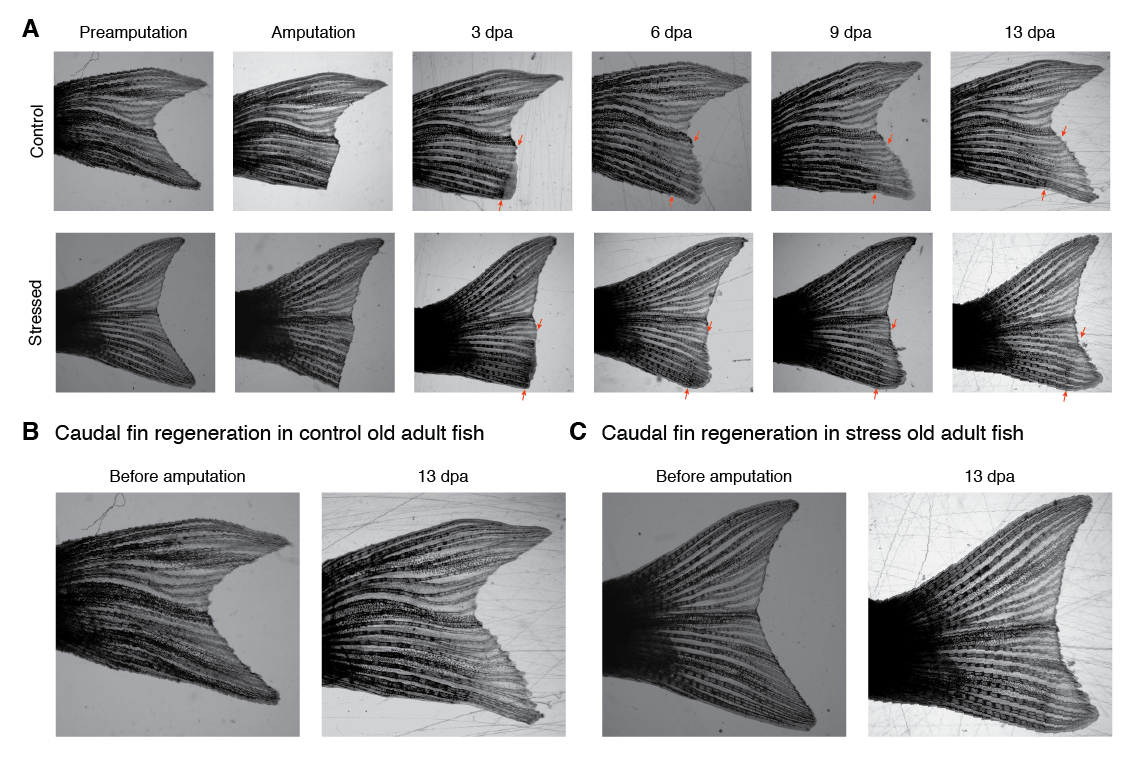


**Figure S4. Irregular tissue growing in stressed old fish.** Caudal fins from a stressed old fish that show bites and signals of aggression after the UCS protocol and before amputation.

**
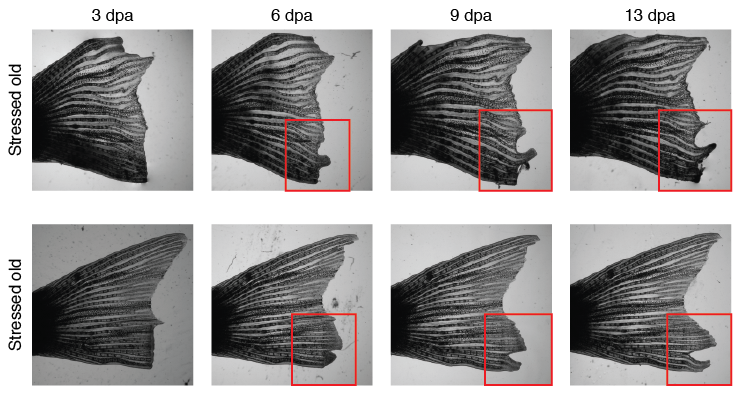
**

**Figure S5. Aggression signals after exposure to the stress protocol in young stressed fish.** A-B-C) Caudal fins from three different young stressed fish that show bites and signals of aggression after the UCS protocol and before amputation. We also show the complete regeneration of the caudal fins, both in the wounds and in the amputation.


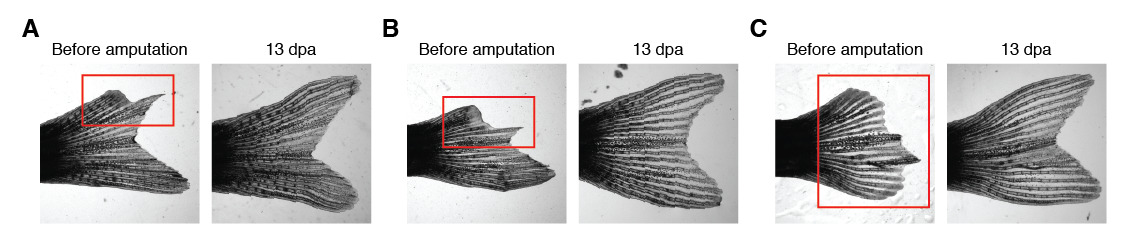

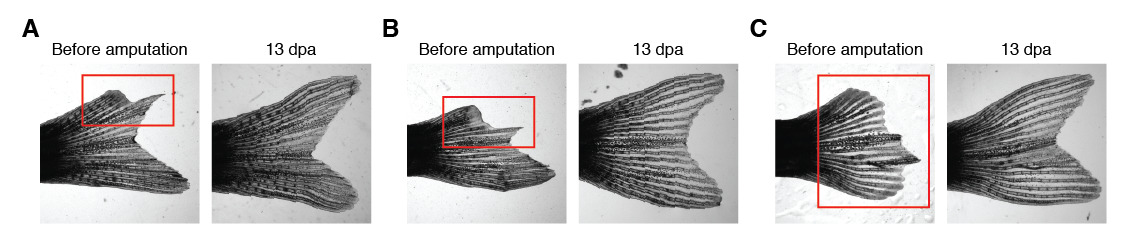

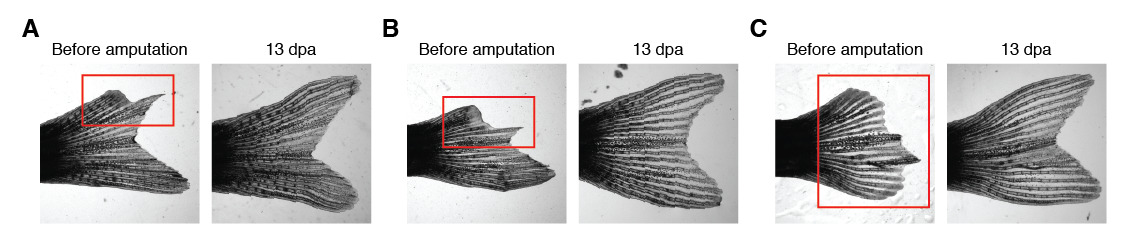


**Figure S6. Visualization of the zebrafish behavior during Open Field test.** Track visualization and heatmap visualization of the swimming patterns of three representative fish from each experimental group (control, stress) in A) young and B) old zebrafish. Rows are the same representative fish during the same recording.


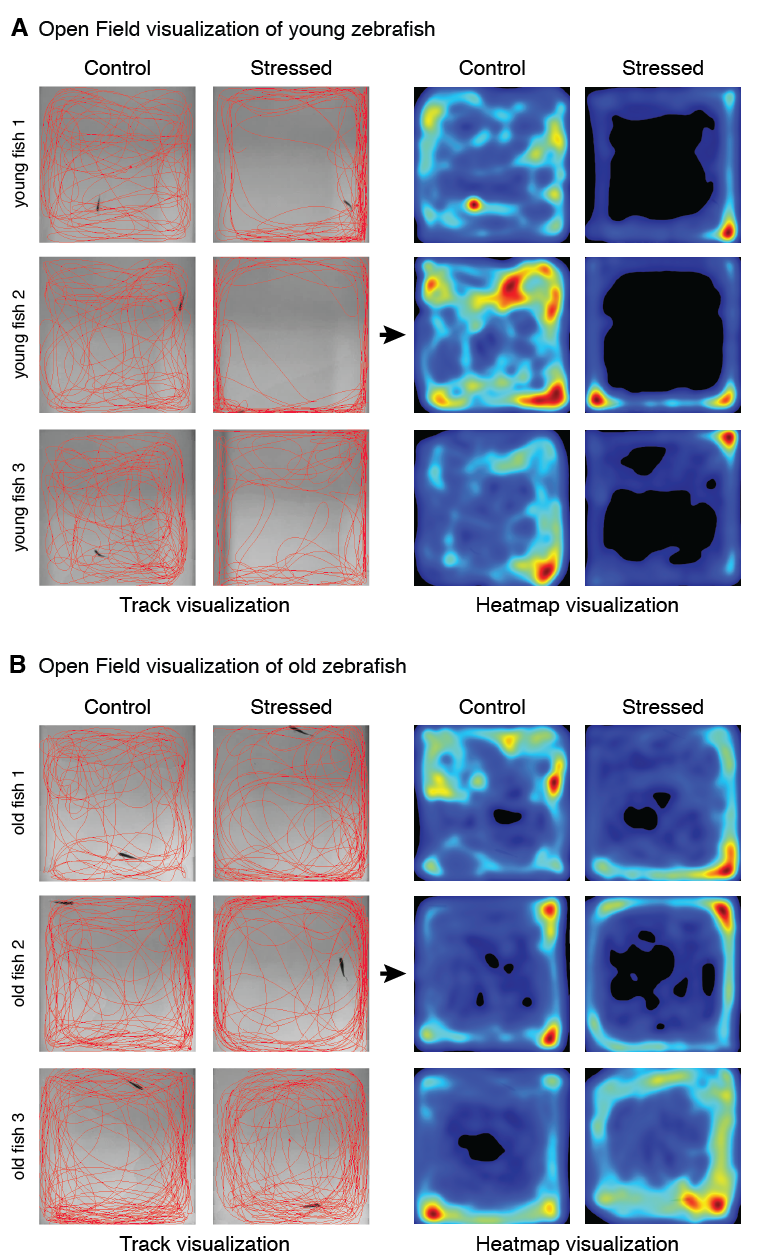


1. ***Supplementary Figures***

**Table S1:** Procedure of the unpredictable chronic stress (UCS) protocol in zebrafish.

- **Cohort 1: old zebrafish (n = 20; control = 10, stress = 10)**

| First eight days of stress protocol | | | | | | | |
| --- | --- | --- | --- | --- | --- | --- | --- |
| ***Day 1*** | ***Day 2*** | ***Day 3*** | ***Day 4*** | ***Day 5*** | ***Day 6*** | ***Day 7*** | ***Day 8*** |
| **11:30 am**  **Stressor (v)** | 10:30 am Stressor (ii) | 9:20 am  Stressor (i) | 11:30 am Stressor (v) | 9:20 am Stressor (ii) | 8:40 am Stressor (i) | 11:30 am Stressor (iv) | 11:00 am  Stressor (iv) |
| **2:00 pm**  **Stressor (vi)** | 1:00 pm Stressor (vii) | 5:00 pm Stressor (iii) | 3:30 pm  Stressor (iii) | 6:00 pm Stressor (vii) | 2:40 pm Stressor (vi) | 4:30 pm  Stressor (vii) | 2:00 pm  Stressor (i) |

| Stress protocol after caudal fin amputation | | | | | | | | | | | | |
| --- | --- | --- | --- | --- | --- | --- | --- | --- | --- | --- | --- | --- |
| ***Day 11*** | ***Day 12*** | ***Day 13*** | ***Day 14*** | ***Day 15*** | ***Day 16*** | ***Day 17*** | ***Day 18*** | ***Day 19*** | ***Day 20*** | ***Day 21*** | ***Day 22*** | ***Day 23*** |
| **9:00 am**  **Stressor (i)** | 9:25 am  Stressor (ii) | 9:45 am Stressor (iii) | 12:14 pm Stressor (v) | 12:00 m Stressor (iv) | 11:30 am Stressor (i) | 7:30 am  Stressor (vii) | 10:30 am  Stressor (vi) | 9:15 am  Stressor (v) | 8:00 am Stressor (vii) | 7:20 am Stressor (ii) | 10:00 am  Stressor (v) | 11:30 am Stressor (iv) |
| **5:00 pm**  **Stressor (vii)** | 2:40 pm Stressor (vi) | **1:00 pm**  **growth record** | 2:30 pm  Stressor (vi) | 2:00 pm Stressor (vii) | **1:00 pm growth record** | 4:00 pm  Stressor (v) | 5:30 pm  Stressor (iv) | **1:00 pm**  **growth record** | 12:45 pm Stressor (iii) | 3:00 pm  Stressor (i) | 5:00 pm Stressor (ii) | **1:00 pm**  **growth record** |

- **Cohort 2: old zebrafish (n = 20; control = 10, stress = 10)**

| First eight days of stress protocol | | | | | | | |
| --- | --- | --- | --- | --- | --- | --- | --- |
| ***Day 1*** | ***Day 2*** | ***Day 3*** | ***Day 4*** | ***Day 5*** | ***Day 6*** | ***Day 7*** | ***Day 8*** |
| 3:00 pm  Stressor (vii) | 3:50 pm Stressor (iii) | 11:10 am  Stressor (vii) | 11:40 am Stressor (i) | 8:30 am Stressor (i) | 8:00 am Stressor (iii) | 3:00 pm Stressor (vii) | 11:50 am  Stressor (iv) |
| 6:50 pm  Stressor (vi) | 7:50 pm Stressor (v) | 4:00 pm Stressor (ii) | 1:30 pm  Stressor (iv) | 4:20 pm Stressor (vi) | 12:00 m Stressor (v) | 4:30 pm  Stressor (ii) | 1:30 pm  Stressor (iii) |

| Stress protocol after caudal fin amputation | | | | | | | | | | | | |
| --- | --- | --- | --- | --- | --- | --- | --- | --- | --- | --- | --- | --- |
| ***Day 11*** | ***Day 12*** | ***Day 13*** | ***Day 14*** | ***Day 15*** | ***Day 16*** | ***Day 17*** | ***Day 18*** | ***Day 19*** | ***Day 20*** | ***Day 21*** | ***Day 22*** | ***Day 23*** |
| 1:20 pm  Stressor (iv) | 8:30 am  Stressor (vii) | 10:00 am Stressor (ii) | 11:40 pm Stressor (iii) | 9:00 m Stressor (v) | 11:00 am Stressor (vi) | 8:30 m Stressor (v) | 10:00 pm Stressor (i) | 9:00 am  Stressor (iii) | 8:00 m Stressor (vi) | 7:30 m Stressor (ii) | 10:00 am  Stressor (iv) | 10:30 am Stressor (i) |
| 2:30 pm  Stressor (i) | 4:00 pm Stressor (vi) | **1:00 pm**  **growth record** | 2:30 pm  Stressor (vii) | 3:00 pm Stressor (vi) | **1:00 pm growth record** | 5:00 pm Stressor (vi) | 2:30 pm  Stressor (vii) | **1:00 pm**  **growth record** | 4:00 pm Stressor (v) | 5:00 pm Stressor (vii) | 5:00 pm Stressor (ii) | **1:00 pm**  **growth record** |

- **Cohort 3: young zebrafish (n = 20; control = 10, stress = 10)**

| First eight days of stress protocol | | | | | | | |
| --- | --- | --- | --- | --- | --- | --- | --- |
| ***Day 1*** | ***Day 2*** | ***Day 3*** | ***Day 4*** | ***Day 5*** | ***Day 6*** | ***Day 7*** | ***Day 8*** |
| 10:00 am  Stressor (v) | 12:30 pm Stressor (iii) | 9:40 am  Stressor (i) | 10:30 am Stressor (vii) | 11:40 am Stressor (iv) | 2:00 pm Stressor (iii) | 12:00 m Stressor (vii) | 10:00 am  Stressor (i) |
| 4:45 pm  Stressor (iv) | 2:50 pm Stressor (ii) | 1:00 pm Stressor (vi) | 11:40 am  Stressor (vi) | 3:40 pm Stressor (i) | 5:00 pm Stressor (v) | 3:00 pm  Stressor (ii) | 2:30 pm  Stressor (vii) |

| Stress protocol after caudal fin amputation | | | | | | | | | | | | |
| --- | --- | --- | --- | --- | --- | --- | --- | --- | --- | --- | --- | --- |
| ***Day 11*** | ***Day 12*** | ***Day 13*** | ***Day 14*** | ***Day 15*** | ***Day 16*** | ***Day 17*** | ***Day 18*** | ***Day 19*** | ***Day 20*** | ***Day 21*** | ***Day 22*** | ***Day 23*** |
| 8:00 am  Stressor (iii) | 9:30 am  Stressor (i) | 8:00 am Stressor (iv) | 11:30 am Stressor (vii) | 4:00 pm Stressor (iii) | 10:00 am Stressor (ii) | 9:30 am Stressor (iv) | 10:00 am Stressor (v) | 9:00 am  Stressor (vii) | 8:00 am Stressor (vi) | 7:30 am Stressor (ii) | 10:00 am  Stressor (i) | 11:30 am Stressor (vii) |
| 2:00 pm  Stressor (vii) | 5:00 pm Stressor (ii) | **1:00 pm**  **growth record** | 12:30 pm  Stressor (v) | 6:00 pm Stressor (vi) | **1:00 pm growth record** | 2:00 pm Stressor (i) | 3:30 pm  Stressor (vi) | **1:00 pm**  **growth record** | 5:00 pm Stressor (iii) | 6:00 pm Stressor (iv) | 4:00 pm Stressor (vi) | **1:00 pm**  **growth record** |

- **Cohort 4: young zebrafish (n = 20; control = 10, stress = 10)**

| First eight days of stress protocol | | | | | | | |
| --- | --- | --- | --- | --- | --- | --- | --- |
| ***Day 1*** | ***Day 2*** | ***Day 3*** | ***Day 4*** | ***Day 5*** | ***Day 6*** | ***Day 7*** | ***Day 8*** |
| 2:00 pm  Stressor (vii) | 11:40 am Stressor (ii) | 10:00 am  Stressor (i) | 11:30 am Stressor (vi) | 10:00 am Stressor (iii) | 8:00 am Stressor (i) | 1:30 pm Stressor (ii) | 10:00 am  Stressor (iv) |
| 2:30 pm  Stressor (vi) | 1:00 pm Stressor (iii) | 4:50 pm Stressor (vii) | 2:30 pm  Stressor (v) | 2:00 pm Stressor (i) | 5:00 pm Stressor (iii) | 3:00 pm  Stressor (vi) | 4:30 pm  Stressor (v) |

| Stress protocol after caudal fin amputation | | | | | | | | | | | | |
| --- | --- | --- | --- | --- | --- | --- | --- | --- | --- | --- | --- | --- |
| ***Day 11*** | ***Day 12*** | ***Day 13*** | ***Day 14*** | ***Day 15*** | ***Day 16*** | ***Day 17*** | ***Day 18*** | ***Day 19*** | ***Day 20*** | ***Day 21*** | ***Day 22*** | ***Day 23*** |
| 11:30 am Stressor (vii) | 7:30 am Stressor (ii) | 10:00 am Stressor (v) | 8:00 am  Stressor (iii) | 11:30 am Stressor (vii) | 10:00 am Stressor (ii) | 9:30 am  Stressor (i) | 8:00 am  Stressor (iii) | 9:00 am  Stressor (vii) | 8:00 am Stressor (vi) | 9:30 am Stressor (iv) | 10:00 am  Stressor (i) | 8:00 am Stressor (iii) |
| 2:30 pm  Stressor (vi) | 6:00 pm Stressor (iv) | **1:00 pm**  **growth record** | 12:30 pm  Stressor (v) | 2:00 pm  Stressor (vii) | **1:00 pm growth record** | 2:00 pm Stressor (i) | 6:00 pm Stressor (vi) | **1:00 pm**  **growth record** | 5:00 pm Stressor (iii) | 5:00 pm Stressor (ii) | 4:00 pm Stressor (vi) | **1:00 pm**  **growth record** |

- **Cohort 5: old zebrafish (n = 8; control = 4, stress = 4)**
- **Cohort 6: young zebrafish (n = 12; control = 6, stress = 6)**

| First eight days of stress protocol | | | | | | | |
| --- | --- | --- | --- | --- | --- | --- | --- |
| ***Day 1*** | ***Day 2*** | ***Day 3*** | ***Day 4*** | ***Day 5*** | ***Day 6*** | ***Day 7*** | ***Day 8*** |
| 10:40 am  Stressor (vii) | 10:40 am Stressor (vi) | 12:00 m  Stressor (ii) | 9:00 am Stressor (i) | 10:00 am Stressor (v) | 9:30 am Stressor (iv) | 11:30 pm Stressor (iii) | 9:45 am  Stressor (v) |
| 5:50 pm  Stressor (i) | 4:50 pm Stressor (iii) | 5:10 pm Stressor (vii) | 2:10 pm  Stressor (v) | 2:00 pm Stressor (iii) | 4:00 pm Stressor (vi) | 4:30 pm  Stressor (vii) | 5:30 pm  Stressor (ii) |

| Stress protocol after caudal fin amputation | | | | | | | | | | | | |
| --- | --- | --- | --- | --- | --- | --- | --- | --- | --- | --- | --- | --- |
| ***Day 11*** | ***Day 12*** | ***Day 13*** | ***Day 14**** | ***Day 15*** | ***Day 16*** | ***Day 17*** | ***Day 18*** | ***Day 19*** | ***Day 20*** | ***Day 21*** | ***Day 22*** | ***Day 23*** |
| 10:20 am Stressor (iii) | 5:00 pm Stressor (iv) | 9:00 am Stressor (vii) | 9:10 am  Stressor (ii) | 11:00 am Stressor (v) | 8:50 am Stressor (iii) | 10:10 am  Stressor (vi) | 10:00 am  Stressor (i) | 10:00 am  Stressor (i) | 10:40 am Stressor (ii) | 11:20 am Stressor (v) | 11:30 am  Stressor (ii) | 11:30 am Stressor (vi) |
| 12:05 pm  Stressor (i) | 7:20 pm Stressor (vi) | **1:00 pm**  **growth record** | 4:30 pm  Stressor (vii) | 5:30 pm  Stressor (i) | **1:00 pm growth record** | 2:50 pm Stressor (iv) | 6:00 pm Stressor (iii) | **1:00 pm**  **growth record** | 12:00 m Stressor (vii) | 3:00 pm Stressor (iii) | 4:00 pm Stressor (iv) | **1:00 pm**  **growth record** |

* Euthanasia of cohort 5 old zebrafish for gene expression test

**Table S2:** Primer design. Target-specific primers for *sam2*, *elf-1*, and *rpl13a* were designed using primer blast and primer quest tools in conjunction with OligoAnalyzer program for the evaluation of the characteristics if each primer. Primers for *nlgn-1* were taken from Krall et al. (2019). These primers pairs amplify short fragments between 70-200 bp, in an exon-exon boundary to avoid amplification of possible contamination with genomic DNA and with Tm close to 60°C, considering a difference in Tm between the pair of primers ≤ 2°C.

| Gen | Forward | Reverse |
| --- | --- | --- |
| *nlgn-1** | GGCTTGGTGTGCTCGGTTTTT | GGTCACCCCCAAAGAAAGCA |
| *sam-2* | TCACATTGTTGAGGTGCGGA | CACCACTTCTGCTGGACGAT |
| *rpl13a* | ACAAAACCAAGAGGGGTCAGG | GACAACCATGCGCTTTCTCTTG |
| *eef1a1l1* | CCAACTTCAACGCTCAGGTCA | CAAACTTGCAGGCGATGTGA |

*Krall et al (2019).

**Table S3:** Results of statistical analyses between age groups (young, old) before and after the stress protocol.

|  |  | t-test young vs old | | | | | | | |
| --- | --- | --- | --- | --- | --- | --- | --- | --- | --- |
|  |  | **Before stress protocol** | | | | **After stress protocol** | | | |
|  |  | **T (df)** | **young young fish**  **(SE)** | **Mean old fish (SE)** | **p-value** | **T (df)** | **young mean (SE)** | **old mean**  **(SE)** | **p-value** |
| Open field |  | **Distance to the center point of the arena** | | | | | | | |
|  | control | 0.05 (26.43) | 8.56 (0.27) | 8.59 (0.40) | 0.96 | 1.94 (33.53) | 8.51 (0.25) | 9.23 (0.27) | **0.061** |
|  | stress | -1.50 (26.56) | 8.79 (0.20) | 8.22 (0.32) | 0.15 | 0.31 (35.91) | 9.22 (0.23) | 9.32 (0.23) | 0.75 |
|  |  | **Total distance traveled** | | | | | | | |
|  | control | 1.98 (23.20) | 1300.23 (58.29) | 1532.025 (101.64) | **0.060** | 1.77 (33.04) | 1255.84 (69.63) | 1454.02 (87.40) | **0.086** |
|  | stress | 1.86 (25.80) | 1353.23 (56.89) | 1557.663 (93.77) | **0.074** | 0.88 (20.99) | 1427.76 (42.30) | 1519.52 (95.11) | 0.39 |
|  |  | **Average velocity** | | | | | | | |
|  | control | 2.00 (23.09) | 4.33 (0.19) | 5.11 (0.34) | **0.057** | 1.78 (30.08) | 4.19 (0.23) | 4.85 (0.29) | **0.085** |
|  | stress | 1.87 (25.62) | 4.51 (0.19) | 5.20 (0.31) | **0.073** | 0.86 (21.11) | 4.76 (0.14) | 5.06 (0.32) | 0.40 |
| Light/dark preference |  | **Time in white zone** | | | | | | | |
|  | control | 1.05 (15.06) | 83.79 (11.84) | 148.90 (61.15) | 0.31 | 0.20 (20.25) | 89.42 (18.67) | 98.07 (39.78) | 0.85 |
|  | stress | 1.61 (19.97) | 52.19 (10.01) | 95.10 (24.73) | 0.12 | 1.01 (32.72) | 55.09 (11.31) | 72.91 (13.60) | 0.32 |
|  |  | **Total number of transitions between zones** | | | | | | | |
|  | control | -4.74 (36.95) | 35.00 (4.17) | 9.60 (3.38) | **<0.001** | -3.015 (36.98) | 35.62 (4.34) | 18.87 (3.47) | **0.005** |
|  | stress | 1.20 (29.10) | 26.25 (3.89) | 34.31 (5.48) | 0.24 | -0.140 (38.00) | 38.33 (6.05) | 37.25 (4.85) | 0.89 |

1. ***Supplementary References***

Antelo-Iglesias, L., Picallos-Rabina, P., Estévez-Souto, V., Da Silva-Álvarez, S., and Collado, M. (2021). The role of cellular senescence in tissue repair and regeneration. Mech Ageing Dev. 198:111528. doi:10.1016/j.mad.2021.111528

Aztekin C. (2021). Appendage regeneration is context dependent at the cellular level. Open Biol. 11(7):210126. doi:10.1098/rsob.210126

Barbazuk, W. B., Korf, I., Kadavi, C., Heyen, J., Tate, S., Wun, E., et al. (2000). The syntenic relationship of the zebrafish and human genomes. Genome Res. 10(9):1351-1358. doi:10.1101/gr.144700

Blaser, R. E., Chadwick, L., and McGinnis, G. C. (2010). Behavioral measures of anxiety in zebrafish (Danio rerio). Behav Brain Res. 208(1):56-62. doi:10.1016/j.bbr.2009.11.009

Blum, N. and Begemann, G. (2013). The Roles of Endogenous Retinoid Signaling in Organ and Appendage Regeneration. Cell Mol Life Sci. 70(20): 3907–27. doi: 10.1007/s00018-013-1303-7.

Bouzaffour, M., Rampon, C., Ramaugé, M., Courtin, F., & Vriz, S. (2010). Implication of type 3 deiodinase induction in zebrafish fin regeneration. Gen Comp Endocrinol. 168(1):88-94. doi:10.1016/j.ygcen.2010.04.006

Collier, A. D., Kalueff, A. V., and Echevarria, D. J. (2017). Zebrafish models of anxiety-like behaviors. In The rights and wrongs of zebrafish: Behavioral phenotyping of zebrafish (pp. 45-72). Springer, Cham. doi: 10.1007/978-3-319-33774-6_3

Da Silva-Álvarez, S., Guerra-Varela, J., Sobrido-Cameán, D., Quelle, A., Barreiro-Iglesias, A., Sánchez, L., et al. (2020). Aging Cell. 19(1):e13052. doi:10.1111/acel.13052

de Wit, L., Fang, J., Neef, K., Xiao, J., A Doevendans, P., Schiffelers, R. M., et al. (2020). Cellular and Molecular Mechanism of Cardiac Regeneration: A Comparison of Newts, Zebrafish, and Mammals. Biomolecules. 10(9):1204. doi:10.3390/biom10091204

Demin, K. A., Lakstygal, A. M., Krotova, N. A., Masharsky, A., Tagawa, N., Chernysh, et al. (2020). Understanding complex dynamics of behavioral, neurochemical and transcriptomic changes induced by prolonged chronic unpredictable stress in zebrafish. Sci Rep. 10(1):19981. doi:10.1038/s41598-020-75855-3

Dreosti, E., Lopes, G., Kampff, A. R., and Wilson, S. W. (2015). Development of social behavior in young zebrafish. 9:39. Published 2015 Aug 18. doi:10.3389/fncir.2015.00039

Facciol, A., Tran, S., and Gerlai, R. (2017). Re-examining the factors affecting choice in the light-dark preference test in zebrafish. Behav Brain Res. 2017;327:21-28. doi:10.1016/j.bbr.2017.03.040

Golla, A., Østby, H., and Kermen, F. (2020). Chronic unpredictable stress induces anxiety-like behaviors in young zebrafish. Sci Rep. 10(1):10339. doi:10.1038/s41598-020-67182-4

Gray, J. D., Rubin, T. G., Hunter, R. G., and McEwen, B. S. (2014). Hippocampal gene expression changes underlying stress sensitization and recovery. 19(11):1171-1178. doi:10.1038/mp.2013.175

Hardy, R. A., Rached, N. A., Jones, J. A., Archer, D. R., and Hyacinth, H. I. (2021). Role of age and neuroinflammation in the mechanism of cognitive deficits in sickle cell disease. Exp Biol Med (Maywood). 2021;246(1):106-120. doi:10.1177/1535370220958011

Hübner, G., Brauchle, M., Smola, H., Madlener, M., Fässler, R., and Werner, S. (1996). Differential regulation of pro-inflammatory cytokines during wound healing in normal and glucocorticoid-treated mice. Cytokine. 8(7):548-556. doi:10.1006/cyto.1996.0074

Iovine M. K. (2007). Conserved mechanisms regulate outgrowth in zebrafish fins. Nat Chem Biol. 3(10):613-618. doi:10.1038/nchembio.2007.36

Krall, M., Htun, S., and Slavotinek, A. (2019). Use of PTC124 for nonsense suppression therapy targeting BMP4 nonsense variants in vitro and the bmp4st72 allele in zebrafish. PloS one, 14(4), e0212121.

Lenze, E. J., and Wetherell, J. L. (2011). A lifespan view of anxiety disorders. Dialogues Clin Neurosci. 2011;13(4):381-399. doi:10.31887/DCNS.2011.13.4/elenze

Mathew, L. K., Sengupta, S., Kawakami, A., Andreasen, E. A., Löhr, C. V., Loynes, C. A., et al. (2007). Unraveling tissue regeneration pathways using chemical genetics. J Biol Chem. 282(48):35202-35210. doi:10.1074/jbc.M706640200

Matthews, M., and Varga, Z. M. (2012). Anesthesia and euthanasia in zebrafish.  ILAR J. 53(2):192-204. doi:10.1093/ilar.53.2.192

Meshalkina, D. A., Kizlyk, M. N., Kysil, E. V., Collier, A. D., Echevarria, D. J., Abreu, M. S., et al. (2017). Understanding zebrafish cognition. Behav Processes. 141(Pt 2):229-241. doi:10.1016/j.beproc.2016.11.020

Miller, N. Y., & Gerlai, R. (2011). Shoaling in zebrafish: what we don't know. Rev Neurosci. 22(1):17-25. doi:10.1515/RNS.2011.004

O'Connor, T. M., O'Halloran, D. J., and Shanahan, F. (2000). The stress response and the hypothalamic-pituitary-adrenal axis: from molecule to melancholia. QJM. 93(6):323-333. doi:10.1093/qjmed/93.6.323

Orger, M. B., and de Polavieja, G. G. (2017). Zebrafish Behavior: Opportunities and Challenges. Annu Rev Neurosci. 40:125-147. doi:10.1146/annurev-neuro-071714-033857

Poss, K. D., Shen, J., Nechiporuk, A., McMahon, G., Thisse, B., Thisse, C., et al. (2000). Roles for Fgf signaling during zebrafish fin regeneration. Dev Biol. 222(2):347-358. doi:10.1006/dbio.2000.9722

Prudovsky I. (2021). Cellular Mechanisms of FGF-Stimulated Tissue Repair. Cells. 10(7):1830. Published 2021 Jul 20. doi:10.3390/cells10071830

Saverino, C., and Gerlai, R. (2008). The social zebrafish: behavioral responses to conspecific, heterospecific, and computer animated fish. Behav Brain Res. 191(1):77-87. doi:10.1016/j.bbr.2008.03.013

Schmidt, J. R., Geurtzen, K., von Bergen, M., Schubert, K., and Knopf, F. (2019). Glucocorticoid Treatment Leads to Aberrant Ion and Macromolecular Transport in Regenerating Zebrafish Fins. Front Endocrinol (Lausanne). 10:674. doi:10.3389/fendo.2019.00674

Sequeira, A., Salas, A., Fornaguera, J., and Brenes, J. C. (2019). Behavioural characterisation of chronic unpredictable stress based on ethologically relevant paradigms in rats. Sci Rep. 9(1):17403. doi:10.1038/s41598-019-53624-1

Shams, S., Chatterjee, D., and Gerlai., R. (2015). Chronic Social Isolation Affects Thigmotaxis and Whole-Brain Serotonin Levels in Adult Zebrafish. Behav Brain Res. 292: 283–87. doi: 10.1016/j.bbr.2015.05.061.

Shams, S., Seguin, D., Facciol, A., Chatterjee, D., and Gerlai, R. (2017). Effect of social isolation on anxiety-related behaviors, cortisol, and monoamines in adult zebrafish Behav Neurosci. 131(6):492-504. doi:10.1037/bne0000220

Song, C., Liu, B. P., Zhang, Y. P., Peng, Z., Wang, J., Collier, et al. (2018). Modeling consequences of prolonged strong unpredictable stress in zebrafish: Complex effects on behavior and physiology. Prog Neuropsychopharmacol Biol Psychiatry. 81:384-394. doi:10.1016/j.pnpbp.2017.08.021

Sorzano, C. O. S., and Parkinson, M. (2019). Statistical experiment design for animal research.

Staab, T. A., Egrafov, O., Knowles, J. A., and Sieburth, D. (2014). Regulation of Synaptic nlg-1/Neuroligin Abundance by the skn-1/Nrf Stress Response Pathway Protects against Oxidative Stress. PLoS Genet. 10(1):e1004100. doi:10.1371/journal.pgen.1004100

Steenbergen, P. J., Richardson, M. K., and Champagne, D. L. (2011). Patterns of avoidance behaviours in the light/dark preference test in young juvenile zebrafish: a pharmacological study. Behav Brain Res. 222(1):15-25. doi:10.1016/j.bbr.2011.03.025

Stewart, A., Gaikwad, S., Kyzar, E., Green, J., Roth, A., and Kalueff, A. V. (2012). Modeling anxiety using adult zebrafish: a conceptual review. Neuropharmacology. 62(1):135-143. doi:10.1016/j.neuropharm.2011.07.037

Stewart, A. M., Braubach, O., Spitsbergen, J., Gerlai, R., and Kalueff, A. V. (2014). Zebrafish models for translational neuroscience research: from tank to bedside. Trends Neurosci. 37(5):264-278. doi:10.1016/j.tins.2014.02.011

Suriyampola, P. S., Shelton, D. S., Shukla, R., Roy, T., Bhat, A., and Martins, E. P. (2016). Zebrafish Social Behavior in the Wild. Zebrafish. 13(1):1-8. doi:10.1089/zeb.2015.1159

Thanapaul, R., Shvedova, M., Shin, G. H., and Roh, D. S. (2021). An Insight into Aging, Senescence, and Their Impacts on Wound Healing. Adv Geriatr Med Res. 3(3):e210017. doi:10.20900/agmr20210017

Whitehead, G. G., Makino, S., Lien, C. L., and Keating, M. T. (2005). fgf20 is essential for initiating zebrafish fin regeneration. Science. 310(5756):1957-1960. doi:10.1126/science.1117637

Wilkinson, H. N., and Hardman, M. J. (2020). Senescence in Wound Repair: Emerging Strategies to Target Chronic Healing Wounds. Front Cell Dev Biol. 8:773. doi:10.3389/fcell.2020.00773

Yun M. H. (2015). Changes in Regenerative Capacity through Lifespan. Int J Mol Sci. 16(10):25392-25432. doi:10.3390/ijms161025392

Zimprich, A., Garrett, L., Deussing, J. M., Wotjak, C. T., Fuchs, H., Gailus-Durner, V., et al. (2014). A robust and reliable non-invasive test for stress responsivity in mice. Front Behav Neurosci. 8:125. doi:10.3389/fnbeh.2014.00125.

Ziv, L., Muto, A., Schoonheim, P. J., Meijsing, S. H., Strasser, D., Ingraham, H. A., et al. (2013). An affective disorder in zebrafish with mutation of the glucocorticoid receptor. Mol Psychiatry. 18(6):681-691. doi:10.1038/mp.2012.64
